# Efficient Inhibition of Human Papillomavirus Infection by L2 Minor Capsid-Derived Lipopeptide

**DOI:** 10.1128/mBio.01834-19

**Published:** 2019-08-06

**Authors:** Huan Yan, Suan-Sin Foo, Weiqiang Chen, Ji-Seung Yoo, Woo-Jin Shin, Christine Wu, Jae U. Jung

**Affiliations:** a Department of Molecular Microbiology and Immunology, Keck School of Medicine, University of Southern California, Los Angeles, California, USA; b Department of Immunology, Hokkaido University Graduate School of Medicine, Sapporo, Japan; Virginia Polytechnic Institute and State University; University of Pittsburgh Medical Center; University of North Carolina at Chapel Hill

**Keywords:** human papillomavirus, L2N lipopeptide, entry inhibitor, minor capsid protein

## Abstract

The amino (N)-terminal region of human papillomavirus (HPV) minor capsid protein (L2) is a highly conserved region which is essential for establishing viral infection. Despite its importance in viral infectivity, the role of the HPV N-terminal domain has yet to be fully characterized. Using fine mapping analysis, we identified a 36-amino-acid (aa) peptide sequence of the L2 N terminus, termed L2N, that is critical for HPV infection. Ectopic expression of L2N with the transmembrane sequence on the target cell surface conferred resistance to HPV infection. Additionally, L2N peptide with chemical or enzymatic lipidation at the carboxyl (C) terminus efficiently abrogated HPV infection in target cells. Among the synthetic L2N lipopeptides, a stearoylated lipopeptide spanning aa 13 to 46 (13-46st) exhibited the most potent anti-HPV activity, with a half-maximal inhibitory concentration (IC_50_) of ∼200 pM. Furthermore, we demonstrated that the 13-46st lipopeptide inhibited HPV entry by blocking *trans*-Golgi network retrograde trafficking of virion particles, leading to rapid degradation. Fundamentally, the inhibitory effect of L2N lipopeptides appeared to be evolutionarily conserved, as they showed cross-type inhibition among various papillomaviruses. In conclusion, our findings provide new insights into the critical role of the L2N sequence in the HPV entry mechanism and identify the therapeutic potential of L2N lipopeptide as an effective anti-HPV agent.

## Abstract (precis)

HPV is a human oncogenic virus that causes a major public health problem worldwide, which is responsible for approximately 5% of total human cancers and almost all cases of cervical cancers. HPV capsid consists of two structure proteins, the major capsid L1 protein and the minor capsid L2 protein. While L2 plays critical roles during the viral life cycle, the molecular mechanism in viral entry remains elusive. Here, we performed fine mapping of the L2 N-terminal region and defined a short 36-amino-acid peptide, called L2N, which is critical for HPV infection. Specifically, L2N peptide with carboxyl-terminal lipidation acted as a potent and cross-type HPV inhibitor. Taken together, data from our study highlight the essential role of the L2N sequence at the early step of HPV entry and suggests the L2N lipopeptide as a new strategy to broadly prevent HPV infection.

## INTRODUCTION

Human papillomavirus (HPV) belongs to the *Papillomaviridae* family, a large group of nonenveloped double-stranded DNA (dsDNA) viruses that infect a wide range of vertebrates, including various mammals, birds, turtles, and snakes (1–5). High-risk HPVs contribute to approximately 5% of total human cancers, being the causative agent of almost all cases of cervical cancer as well as many other anogenital and oropharyngeal carcinomas (6, 7). Among all the types of HPV, HPV16 is one of the oncogenic types that have been commonly used in HPV research (8). The capsid of HPV consists of two structural proteins, the major capsid protein (L1) and the minor capsid protein (L2). The L1 proteins form pentamers that take up the majority of the capsid surface, whereas L2 proteins are predominantly buried in the capsid, exposing the N-terminal residues on the surface upon cell attachment (9). In fact, L2 is essential for establishing viral infectivity but not for viral capsid assembly. Despite the significance of L2 in facilitating HPV infection, its molecular mechanism in viral entry has not been fully characterized (10, 11).

HPV infection is first initiated by L1-mediated capsid attachment to the extracellular matrix (ECM) and cell surface through heparan sulfate proteoglycan (HSPG) (12–14). This interaction leads to conformational changes of the capsid, facilitated by cyclophilin B (CYPB) (15) and kallikrein-8 (KLK8) (16). This conformational change subsequently enables furin cleavage of the L2 N-terminal region (17, 18), giving rise to the exposure of an N-terminal neutralization epitope that can be recognized by RG1 antibody (amino acids [aa] 17 to 36) or JWW-1 antibody (aa 18 to 32) (19–21). These sequential conformational changes ultimately lead to a reduced affinity between capsid and HSPG, which facilitates capsid transfer to a yet poorly characterized entry receptor(s), thereby promoting endocytosis (21, 22). The uncoating of HPV capsid occurs in multivesicular endosomes (MVEs), in which endosomal acidification and cyclophilin B aid the dissociation of L1 from the L2/viral genome complex. Finally, while a majority of L1 traffics to lysosomes for degradation, a small portion of L1 accompanies the L2/DNA complex throughout the entry process (23).

L2 is a versatile protein that participates in a series of downstream subcellular trafficking events to ensure the nuclear delivery of the HPV genome. Increasing evidence suggests that L2 protein protrudes and spans across the endosomal membrane to interact with a variety of cell-sorting factors, such as the retromer complex, sorting nexins, and dynein through its carboxyl (C)-terminal cytosolic region (aa 68 to 473) (24–27). The L2/DNA complex egresses from the endosome, retrograde traffics to the *trans*-Golgi network (TGN), and ultimately enters the nucleus in a mitosis-dependent manner (26, 28–32). Recent studies have suggested how L2 achieves its transmembrane topology (33–36). The C-terminal cell penetration peptide (cpp) of L2 that protrudes through the endosomal membrane initiates membrane insertion, while its N-terminal putative transmembrane (TM) domain (aa 13 to 46) then reaches the membrane bilayer. γ-Secretase has been proposed as a chaperone that interacts with the L2 TM domain to promote its membrane insertion, where the N-terminal region upstream of the TM remains in the lumen throughout the entry process after insertion (37).

Sequence alignment indicates that the region spanning L2 N-terminal aa 13 to 78 of HPV is highly conserved. This region contains a furin cleavage sequence (RTKR) (17), an RG1/JWW-1 cross-type neutralization epitope with the conserved cysteine residues for intramolecular disulfide bonds (17, 38), and a putative TM domain adjacent to the glycine-rich GXXXG motif (34). However, its role(s) in HPV infection has not been fully characterized. Here, our extensive mutational analysis of this L2 N-terminal region defined the critical residues for HPV infection. We also identified the short L2N 36-amino-acid peptide as a specific and potent inhibitor of HPV infection. In addition, the ectopic surface expression of this L2N peptide abrogated the infectivity of various types of HPV, indicating that the L2N peptide may target an evolutionarily conserved trafficking step that is critical for HPV entry. Together, data from our study highlight a critical role of the L2 N-terminal region for HPV entry and suggest a potential therapeutic strategy for efficient and broad-spectrum inhibition of HPV infection.

## RESULTS

### Fine mapping of the L2 N-terminal conserved region critical for HPV infectivity.

**FIG 1:**
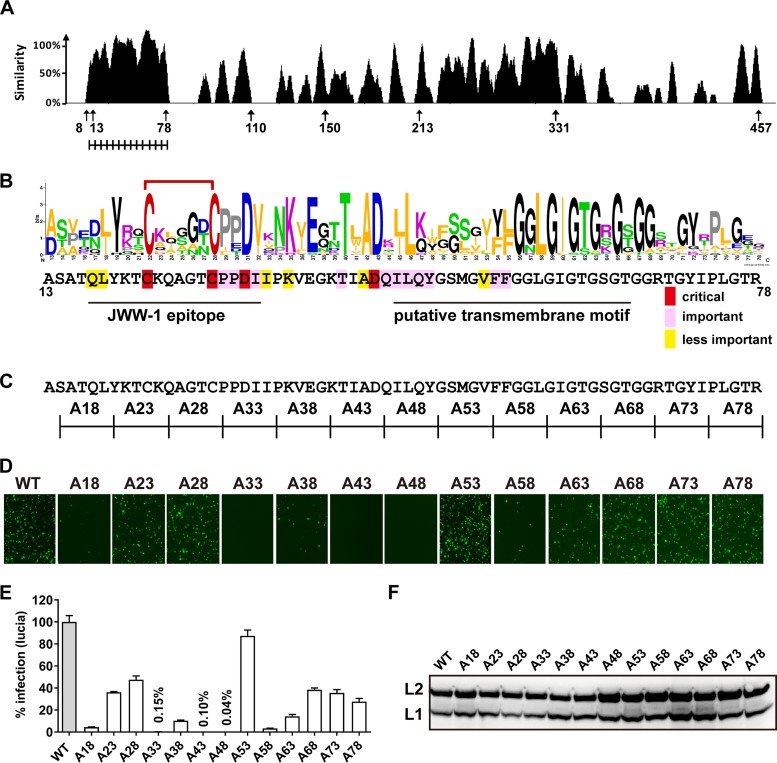
Fine mapping of the L2 N-terminal conserved region critical for HPV infectivity. (A) L2 similarity graph generated by using Vector NTI software, based on L2 sequences from 118 types of HPV (numbered according to HPV16 L2). The N-terminal region spanning aa 13 to 78 was selected for an alanine-scanning substitution assay. (B) L2 residue frequency graph generated by using WebLogo online software, based on aa 13 to 78 of L2 sequences from 118 types of HPV. The contribution of these residues to HPV infectivity (bottom) was summarized from HPV L2 fine mapping results in Fig. S2 and Table S1 in the supplemental material. (C) Illustration of 13 different L2 mutants (A18, A23, A28, A33, A38, A43, A48, A53, A58, A63, A68, A73, and A78) with sequential 5-amino-acid alanine substitutions. C_20_ and C_28_ were not changed in mutants A23 and A28. (D to F) CHO-K1 cells were infected with HPV-GFP/Lucia carrying L2 mutants. (D and E) GFP intensity (D) and Lucia activity (E) of infected cells were analyzed at 36 hpi. (F) L1 and L2 protein levels of input viruses were evaluated by using anti-HPV16 L1 (MD2H11) and anti-HPV16 L2 (2JGmab#5), respectively.

The alignment of the L2 sequences from 118 types of HPV showed considerably high conservation at the N-terminal aa 13 to 78 (numbered based on the HPV16 sequence) (Fig. 1A; see also Fig. S1 in the supplemental material). Using an amino acid substitution mapping strategy, we screened for residues which are critical for HPV infectivity. Briefly, HPV16 pseudovirus (PsV) carrying green fluorescent protein (GFP) or Lucia was assembled by wild-type L1 (L1 WT) along with L2 WT or its mutants, and HPV infection assays were conducted in CHO-K1 cells (39). Two cysteines (C_22_ and C_28_) were not changed due to their essential role in HPV infectivity (Fig. 1C) (38, 40). Among 13 different mutants with 5-aa alanine-scanning substitutions, the A18, A38, and A58 mutants displayed 3 to 20% of the infectivity of L2 WT, while the A33, A43, and A48 mutants almost lost their infectivity. Other mutants (A23, A28, A53, A63, A68, A73, and A78) showed levels of infectivity similar to that of L2 WT (Fig. 1D and E). L2 WT and mutants were expressed at equivalent levels (Fig. 1F). Additional mutational analyses, including 3-aa alanine-scanning (3A), polyglycine-scanning (G), and lysine (K) substitutions (Fig. S2 and Table S1), were also included for HPV PsV infectivity assays. Taken together, our data demonstrate that besides C_22_ and C_28_ for the disulfide bridge and F_54_ and F_55_ in the transmembrane region, two additional aspartic acids (D_31_ and D_43_) and their adjacent conserved residues in the L2 N-terminal region are also critical for HPV PsV infection (Fig. 1B).

10.1128/mBio.01834-19.1 FIG S1 Alignment of L2 N-terminal aa 13 to 75 of 118 types of HPV. Sequence alignment of aa 13 to 75 of L2 proteins from 118 types of HPV was generated by using Vector NTI software. Download FIG S1, JPG file, 2.7 MB. Copyright © 2019 Yan et al. 2019 Yan et al. This content is distributed under the terms of the Creative Commons Attribution 4.0 International license.

10.1128/mBio.01834-19.2 FIG S2 L2 N-terminal fine mapping through 3-aa alanine-scanning (3A), polyglycine-scanning (G), and lysine (K) substitutions. (A) CHO-K1 cells were infected with HPV-Lucia with the indicated mutation in the L2 N-terminal region. Lucia activity in the supernatants was determined at 36 hpi. The relative infectivity of the mutants is shown as a percentage of infection compared with WT HPV16. (B) L1 and L2 protein levels of different mutant viruses were analyzed by immunoblotting with antibodies against HPV16 L1 (MD2H11) and L2 (2JGmab#5). Download FIG S2, JPG file, 1.9 MB. Copyright © 2019 Yan et al. 2019 Yan et al. This content is distributed under the terms of the Creative Commons Attribution 4.0 International license.

10.1128/mBio.01834-19.7 TABLE S1 Plasmids used to produce wild-type and mutant HPVs or other PVs. (A) Plasmids for expressing L1/L2 proteins form different PVs and reporter plasmids for PsV encapsidation. (B) Plasmids for the HPV L2 N-terminal fine mapping assay and the corresponding infectivity of HPV PsV L2 mutants. Download Table S1, PDF file, 0.05 MB. Copyright © 2019 Yan et al. 2019 Yan et al. This content is distributed under the terms of the Creative Commons Attribution 4.0 International license.

### Ectopic expression of the L2 N-terminal region blocks HPV infection.

**FIG 2:**
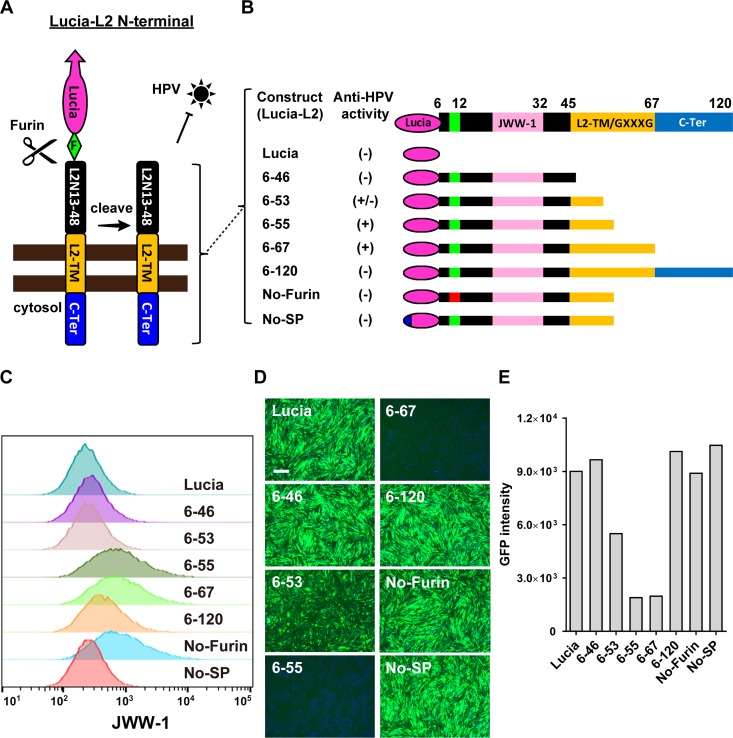
Ectopic expression of the L2 N-terminal region blocks HPV infection. (A) Schematic diagram of the Lucia-L2 N-terminal region fusion construct. F, furin cleavage sequence (RTKR); TM, transmembrane domain; C-ter, C-terminal region. (B) Illustration of the Lucia fusion constructs carrying different L2 N-terminal fragments and their anti-HPV activity. (+), >90% inhibition; (−), <10% inhibition; (+/−), 10 to 90% inhibition; No-Furin, R_9_K and R_12_K mutations in the furin cleavage sequence; No-SP, no secretion signal peptide. (C) FACS analysis of surface expression of Lucia-L2 N-terminal region fusion constructs in CHO-K1 cells with JWW-1 antibody. (D and E) HPV-GFP infection in CHO-K1 cells expressing the indicated Lucia-L2 N-terminal region fusion constructs. (D) Images were captured at 36 hpi. Bar, 200 μm. (E) Total GFP intensity was analyzed by using ImageJ.

A previous study indicated that furin-cleaved L2 achieves a membrane-spanning topology in the endosome (41). To test whether ectopic expression of this L2 N-terminal region on the cell surface affected HPV infection, we constructed various lengths of the L2 N-terminal region (including a furin cleavage sequence at aa 8 to 12) fused with the secretion carrier Lucia, a secreted luciferase (InvivoGen) (Fig. 2A). As negative controls, we constructed an L2 protein carrying the R_9_K and R_12_K mutations at the furin cleavage site (No-Furin) and also a construct without the secretion signal peptide (No-SP) (Fig. 2B). These N-terminal region fusion constructs were stably expressed in CHO-K1 cells by lentivirus transduction, followed by measuring the infection efficiency of HPV PsV carrying a GFP reporter (HPV-GFP). An extracellular Lucia enzymatic assay indicated equivalent expression levels of these Lucia-L2 N-terminal fusion constructs (Fig. S3). Flow cytometry with anti-JWW-1 antibody showed high surface expression levels of the aa 6 to 55 (6-55), 6-67, and No-Furin constructs; intermediate surface expression of the 6-120 construct; and low surface expression levels of the 6-46, 6-53, and No-SP constructs (Fig. 2C). This indicates that a minimal L2-TM sequence is required for efficient surface expression. Consistent with their surface expression, the 6-55 and 6-67 constructs carrying a partial or full-length putative TM domain effectively blocked HPV-GFP infection, while the rest of the constructs did not inhibit infection (Fig. 2D and E). Indeed, no inhibition was detected with the No-Furin and No-SP constructs, indicating that both cell surface expression and furin cleavage are essential for inhibitory activity. On the other hand, the strong inhibitory activity of the 6-67 construct was also observed in CHO-K1, pgsA-745, and U251 cells but not in HeLa, HaCaT, and 293T cells, suggesting a possible cell type-specific inhibition mechanism of the L2 N-terminal region (Fig. S4). Overall, these results demonstrate that the surface display of the L2 N-terminal region efficiently blocks HPV infection in a cell type-dependent manner.

10.1128/mBio.01834-19.3 FIG S3 Lucia activity in the supernatants of CHO-K1 cells expressing Lucia-L2 N-terminal-region fusion constructs. CHO-K1 cells were transduced by lentivirus expressing the indicated Lucia-L2 N-terminal region fusion proteins. A Lucia assay was conducted 3 days after transduction with 10 μl of the supernatant. Download FIG S3, TIF file, 1.6 MB. Copyright © 2019 Yan et al. 2019 Yan et al. This content is distributed under the terms of the Creative Commons Attribution 4.0 International license.

10.1128/mBio.01834-19.4 FIG S4 Anti-HPV activity of Lucia-L2N-6-67 in different cell types. The indicated cells were transduced by lentivirus expressing Lucia-L2N-6-67 and selected with puromycin. The transduced cells were infected with HPV-GFP at 3 days postselection. Images were captured at 48 hpi. Bars, 100 μm. Download FIG S4, JPG file, 2.7 MB. Copyright © 2019 Yan et al. 2019 Yan et al. This content is distributed under the terms of the Creative Commons Attribution 4.0 International license.

### Surface display of L2 N-terminal aa 13 to 48 with a surrogate transmembrane domain efficiently blocks HPV infection.

**FIG 3:**
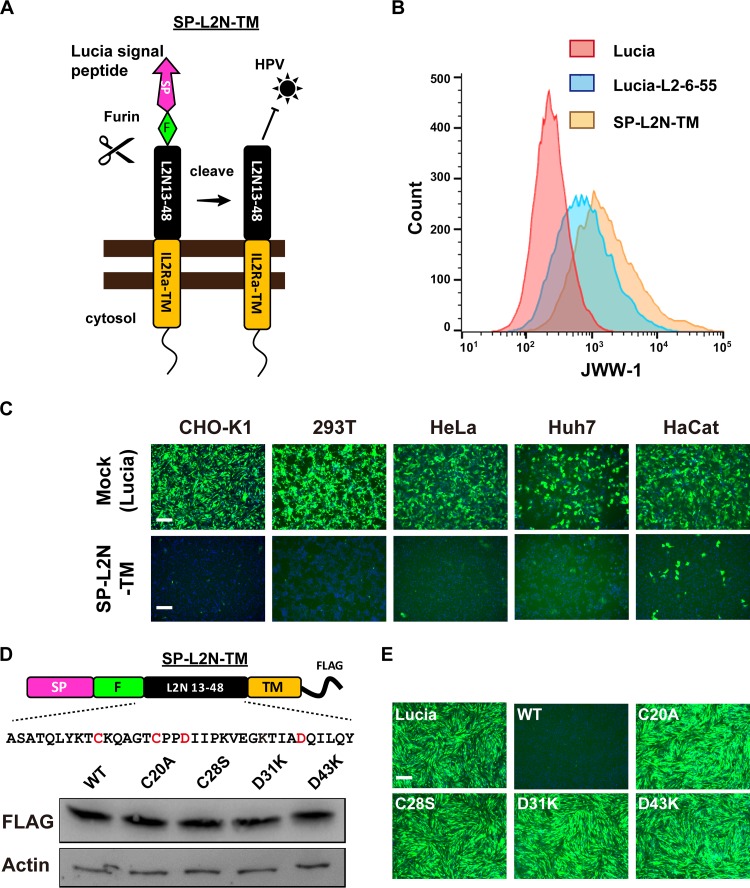
Surface display of L2N aa 13 to 48 with the IL-2Rα transmembrane domain efficiently blocks HPV infection. (A) Schematic diagram of the secretion signal peptide (SP)-L2N-TM construct for surface display of L2 N-terminal aa 13 to 48. Tag, C-terminal tag. (B) Surface expression of the L2 N-terminal region on CHO-K1 cells stably expressing Lucia, Lucia-L2-6-55, or SP-L2N-TM. Surface expression levels were analyzed by flow cytometry with JWW-1 antibody. (C) Different cell lines expressing Lucia (mock) or SP-L2N-TM were infected by HPV-GFP. GFP intensity was analyzed at 36 hpi. Bars, 200 μm. (D) Expression levels of SP-L2N-TM mutants. (Top) Schematic diagram demonstrating the sequences of L2N aa 13 to 48 and highlighting the residues for mutagenesis analysis. (Bottom) Expression levels of SP-L2N-TM mutants in CHO-K1 cells at 3 days posttransduction were evaluated by immunoblotting with anti-FLAG antibody. (E) CHO-K1 cells expressing the indicated SP-L2N-TM mutants were infected with HPV-GFP. GFP intensity was analyzed at 36 hpi. Bar, 200 μm.

We hypothesized that the cell type-specific phenotype might be attributed to the membrane-tethering efficiency of the L2 TM-like motif, as it does not carry an authentic membrane-spanning sequence. To test this hypothesis, SP-L2N-TM was constructed, where the L2 N-terminal aa 13 to 48 were fused with the interleukin-2 receptor alpha subunit (IL-2Rα) TM domain along with the Lucia signal peptide (Fig. 3A). This SP-L2N-TM construct was efficiently expressed on the cell surface, as demonstrated by flow cytometry analysis with JWW-1 antibody (Fig. 3B). Consistent with its high level of surface expression, SP-L2N-TM effectively and broadly blocked HPV-GFP infection in CHO-K1, HeLa, Huh7, 293T, and HaCaT cells (Fig. 3C). Further mutagenesis analysis showed that the C_20_A, C_28_S, D_31_K, or D_43_K mutation of SP-L2N-TM abolished its anti-HPV activity (Fig. 3D and E). This suggests that the transmembrane topology and several specific sequences of L2N are critical for anti-HPV activity.

### Inhibition of HPV infection by L2N lipopeptides.

**FIG 4:**
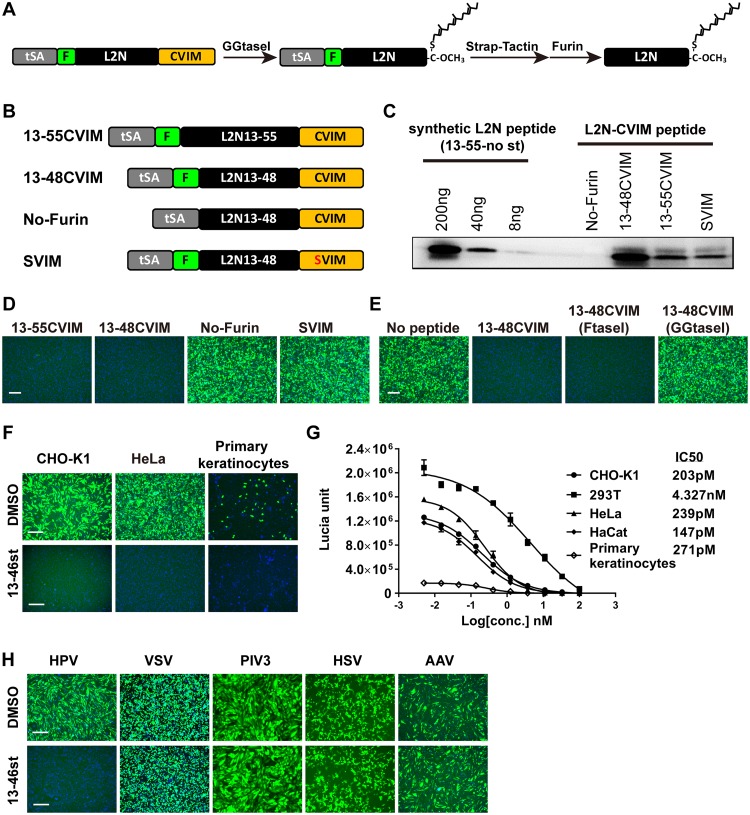
Inhibition of HPV infection by L2N lipopeptides. (A) Overview of L2N-CVIM peptide production. tSA, Twin-Strep-tag; F, furin cleavage site; CVIM, CAAX motif. (B) Schematic diagram of L2N-CVIM constructs. No-Furin, no furin cleavage sequence; SVIM, cysteine-to-serine mutation in the CVIM motif. (C) Quantification of L2N lipopeptides by JWW-1 immunoblotting with different amounts of a synthetic L2N peptide (13-55-no st). (D) HeLa cells were infected with HPV-GFP and treated with the indicated lipopeptides (∼500 nM) during inoculation. GFP intensity was analyzed at 36 hpi. Bar, 200 μm. (E) 13-48CVIM peptides were produced in 293T cells treated with 1 μM FTase inhibitor II (FtaseI) and 1 μM GGTase I inhibitor 2133 (GGtaseI) at 4 h posttransfection. The anti-HPV activity of these peptides was tested by an HPV-GFP infection assay in HeLa cells by coincubation with the inoculum for 16 h at a concentration of ∼500 nM. GFP intensity was analyzed at 36 hpi. Bar, 200 μm. (F) Inhibition of HPV-GFP infection by the 13-46st peptide (200 nM) in CHO-K1 cells, HeLa cells, and primary human epidermal keratinocytes. Bars, 200 μm. (G) IC_50_s of 13-46st peptide in different cells. Cells were infected with HPV-Lucia and treated with different concentrations of 13-46st during inoculation. Lucia activity was determined at 36 hpi (*n* = 2). IC_50_s were calculated by using GraphPad Prism software. (H) Cells were infected with GFP-carrying VSV, PIV3, HSV, or AAV at an MOI of 1 with or without treatment with 1 μM 13-46st during inoculation. Infection assays were conducted in CHO-K1 cells, except for HSV infection, which was conducted in Vero cells. GFP intensity was examined at 24 to 48 hpi. Bars, 100 μm.

Next, we tested whether C-terminal lipidation of the L2N peptide could mimic its transmembrane domain function. We first utilized cysteine-aliphatic-aliphatic-undefined amino acid (CAAX) motif-mediated posttranslational isoprenylation (42) to achieve C-terminal lipidation of L2N. The N-terminal Twin-Strep-tag (tSA) (43) and furin cleavage site (F) were included for peptide purification, and the C-terminal CVIM motif was employed for the isoprenylation modification (Fig. 4A). Furin cleavage sequence deletion (No-Furin) and the Cys-to-Ser (SVIM) mutation were included as negative controls (Fig. 4B). The 13-55CVIM and 13-48CVIM peptides were purified by Twin-Strep-tag pulldown and eluted by furin digestion, followed by immunoblot quantitation with JWW-1 antibody (Fig. 4C). Both the 13-48CVIM and 13-55CVIM peptides suppressed HPV-GFP infection. In contrast, the furin cleavage sequence deletion (No-Furin) or the SVIM mutation completely abolished their ability to inhibit HPV-GFP infection (Fig. 4D). Since CAAX-mediated isoprenylation is achieved by either farnesyltransferase (FTase) or geranylgeranyltransferase type I (GGTase I) (42), the 13-48CVIM peptide was purified from 293T cells in the presence of an FTase inhibitor or a GGTase I inhibitor and tested for anti-HPV activity. Interestingly, treatment with the GGTase I inhibitor, but not the FTase inhibitor, during peptide production led to a loss of anti-HPV activity of the 13-48CVIM peptide (Fig. 4E). This result suggests that the 20-carbon geranylgeranylation by GGTase I is the major form of 13-48CVIM modification. Finally, additional deletion or point mutation analysis showed that aa 13 to 46 were sufficient to achieve strong anti-HPV activity. Hence, this minimal sequence not only is critical for HPV infection but also is required for the anti-HPV activity of the 13-48CVIM peptide (Fig. S5 and Table S2).

10.1128/mBio.01834-19.5 FIG S5 Fine mapping results for L2N-CVIM lipopeptides. (A) Illustrations of deletions (top) and mutations (bottom) of L2N-CVIM lipopeptides. The pink background indicates the regions important for peptide activity. “−” indicates deletions. (B and C) CHO-K1 cells were infected with HPV-Lucia in the presence of the indicated peptides (about 500 nM). The WT peptide was used at four different concentrations to compare its anti-HPV potency with those of other mutant peptides. Lucia activity of infected cells was evaluated at 36 hpi. Download FIG S5, TIF file, 1.9 MB. Copyright © 2019 Yan et al. 2019 Yan et al. This content is distributed under the terms of the Creative Commons Attribution 4.0 International license.

10.1128/mBio.01834-19.8 TABLE S2 Plasmids for expressing L2N fusion proteins or L2N-CVIM peptides. (A) Protein and nucleotide sequences of motifs and affinity tags used in this study. (B) Plasmids for lentiviral production and stable expression of L2 N-terminal region fusion constructs for cell surface display and their anti-HPV activities. (C) Plasmids to produce tSA-L2N-CVIM lipopeptides and their anti-HPV activity. Download Table S2, PDF file, 0.05 MB. Copyright © 2019 Yan et al. 2019 Yan et al. This content is distributed under the terms of the Creative Commons Attribution 4.0 International license.

Alternatively, synthetic L2N peptides were generated with a stearoylation (C_18_) at the C terminus along with a C_22_-C_28_ disulfide bridge. Synthetic L2N peptides without stearoylation or with the D_43_K mutation were synthesized as negative controls. The IC_50_ of these stearoylated L2N lipopeptides was determined for the inhibition of HPV-Lucia infectivity (Table 1). The stearoylated lipopeptides spanning aa 13 to 55 (13-55st), aa 13 to 50 (13-50st), and aa 13 to 46 (13-46st) were highly potent in inhibiting HPV-Lucia infection. In contrast, the L2N peptide with no stearoylation (13-55-no st) showed almost no HPV inhibition activity, and the stearoylated L2N peptide with the D_43_K mutation (13-50st-D43K) showed significantly reduced anti-HPV activity (Table 1). Consistent with the L2N-CVIM peptide mapping results (Fig. S5), the synthetic 13-46st peptide showed the strongest anti-HPV activity in various cell types, including CHO-K1, 293T, HeLa, and HaCaT cells and primary human epidermal keratinocytes, with IC_50_s close to 200 pM (Fig. 4F and G). Finally, the antiviral activity of 13-46st was tested for several other mammalian viruses, including vesicular stomatitis virus (VSV)-pseudotyped lentivirus, human parainfluenza virus 3 (hPIV3), herpes simplex virus (HSV), and adeno-associated virus (AAV). None of viruses were inhibited by the synthetic 13-46st lipopeptide even at a concentration of 1 μM, indicating its specific anti-HPV activity (Fig. 4H). Collectively, these results demonstrated that the L2N lipopeptide spanning aa 13 to 46 potently and specifically blocks HPV infection.

**TABLE 1:** Sequence and anti-HPV activity of synthetic L2N peptides

Peptide	Amino acid sequence^*a*^	Disulfide	Modification(s)	IC_50_^*b*^
JWW-1	LYKTCKQAGTCPPDI	C_20_-C_28_	None	ND
13-55-no st	ASATQLYKTCKQAGTCPPDIIPKVEGKTIADQILQYGSMGVFF	C_20_-C_28_	None	>1 μM
13-55st	ASATQLYKTCKQAGTCPPDIIPKVEGKTIADQILQYGSMGVFFK(stearic acid)	C_20_-C_28_	Stearoylation	100 nM
13-50st	ASATQLYKTCKQAGTCPPDIIPKVEGKTIADQILQYGSK(stearic acid)	C_20_-C_28_	Stearoylation	30 nM
13-50st-D43K	ASATQLYKTCKQAGTCPPDIIPKVEGKTIAKQILQYGSK(stearic acid)	C_20_-C_28_	Stearoylation	200 nM
13-46st	ASATQLYKTCKQAGTCPPDIIPKVEGKTIADRILGTK(stearic acid)	C_20_-C_28_	Stearoylation	<1 nM
FITC-m13-46st	DSASNLYRQCQVTGNCPPDVVNKVEGNTLADRILGSK(FITC)K(stearic acid)	C_20_-C_28_	Stearoylation, FITC	<10 nM

a Cysteines for disulfide bridges are underlined.

b CHOK1 cells were infected by HPV-Lucia and treated with different concentrations of peptides during inoculation. Lucia activity was analyzed at 36 hpi for IC_50_ determination. ND, not detected.

### The L2N lipopeptide blocks HPV entry without affecting HPV capsid priming and internalization.

**FIG 5:**
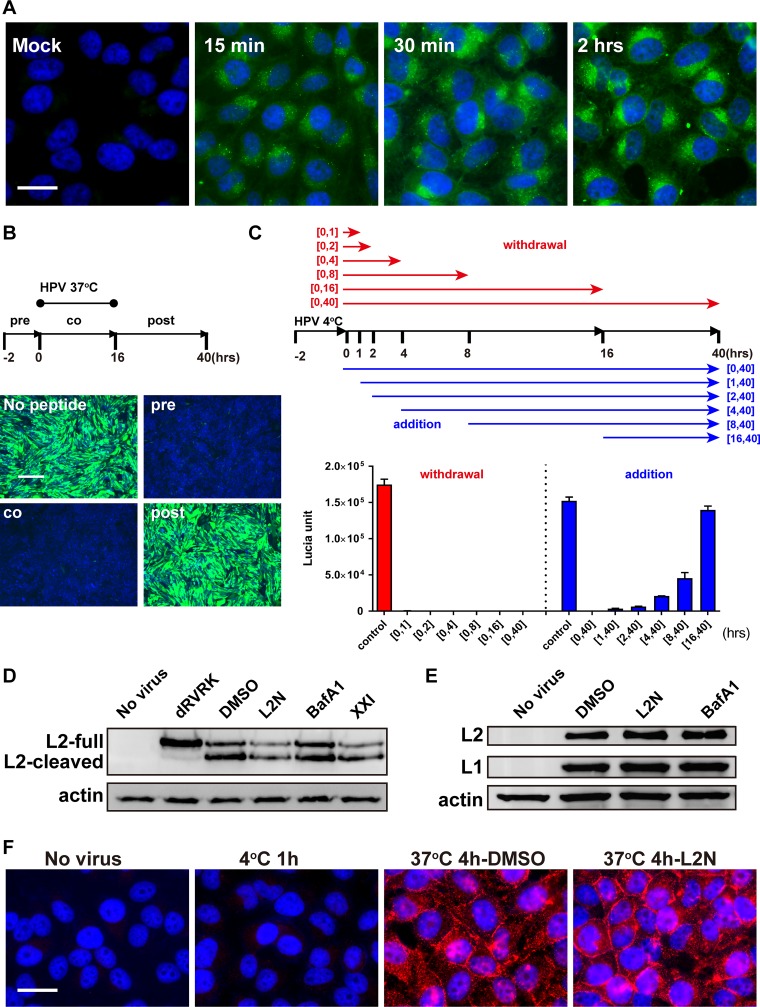
The L2N lipopeptide blocks HPV entry without affecting HPV capsid priming and internalization. (A) Cell binding and uptake of L2N lipopeptide. HeLa cells were incubated with 500 nM FITC-m13-46st peptide for indicated times. Images were captured after three PBS washes. Bar, 25 μm. (B) L2N lipopeptide inhibits HPV-GFP entry. HeLa cells were treated with 500 nM L2N lipopeptide (13-46st) before (pre), during (co), or after (post) HPV-GFP inoculation. Mock, no peptide treatment. GFP intensity was analyzed at 36 hpi. Bar, 200 μm. (C) Peptide addition/withdrawal assays were performed as indicated in the schematic diagram (top). CHO-K1 cells were incubated with HPV-Lucia at 4°C for 2 h. The 13-46st lipopeptide (500 nM) was subsequently added or withdrawn from the medium 1, 2, 4, 8, and 16 h after viral attachment. Lucia activity was analyzed at 40 hpi. (D) CHO-K1 cells were infected with HPV-L2-PSTCD-3×FLAG (MOI of 50) and treated with the indicated chemicals: 500 nM dRVRK, 500 nM L2N lipopeptide 13-46st (L2N), 100 nM BafA1, and 1 μM γ-secretase inhibitor XXI. L2 immunoblotting was performed with anti-FLAG antibody at 16 hpi. (E) CHO-K1 cells were infected by HPV-GFP (MOI of 50) and treated with 500 nM L2N lipopeptide 13-46st, 100 nM BafA1, or both for 2 h. Intracellular L1 and L2 levels were determined by immunoblotting with MD2H11 and JWW-1, respectively. (F) HeLa cells were incubated with HPV-GFP (MOI of 50) at 4°C for 1 h and then shifted to 37°C for 4 h with or without 500 nM FITC-m13-46st. HPV L2 JWW-1 epitope exposure was analyzed by immunostaining with JWW-1 antibody (in red). Bar, 10 μm.

Fluorescein isothiocyanate (FITC)-conjugated 13-46st lipopeptide derived from the Mus musculus papillomavirus 1 (MmuPV1) L2 protein (FITC-m13-46st) retained strong anti-HPV activity (Table 1). To investigate how the L2N lipopeptide blocked HPV infection, we first employed FITC-m13-46st lipopeptide to trace peptide binding and localization. Cell binding and uptake of the L2N lipopeptide were readily detected on HeLa cells at 15 min postincubation, and significant internalization was observed at 30 min postincubation and became apparent at 2 h postincubation (Fig. 5A). To test whether the L2N lipopeptide bound to cell-attached or internalized HPV virion particles to block infection, cells were infected with 5-ethynyl-20-deoxyuridine (EdU)-labeled HPV in the presence of FITC-m13-46st peptide. The localization of HPV virions was detected by a click reaction between Alexa 555 azide and EdU-incorporated HPV DNA. The result demonstrated no significant colocalization between the FITC-m13-46st lipopeptide and HPV DNA at 4 h postinfection (hpi) (Fig. S6), suggesting that the L2N stearic acid-based lipopeptide does not bind to HPV particles to block infection. Furthermore, when the 13-46st lipopeptide was added at the pre-, co-, or postincubation step, HPV-GFP infection was blocked by either pretreatment or coincubation with the 13-46st lipopeptide but not by postincubation with the 13-46st lipopeptide (Fig. 5B). Subsequently, we conducted peptide addition/withdrawal assays to further examine the time window of the L2N lipopeptide action. CHO-K1 cells were first incubated with HPV-Lucia at 4°C for 2 h to allow viral attachment, and the 13-46st lipopeptide was added and/or withdrawn from the medium 1, 2, 4, 8, and 16 h after viral attachment. An “addition” assay showed that the anti-HPV efficiency dramatically decreased when the L2N lipopeptide was applied 4 h or 16 h after viral attachment. A “withdrawal” assay showed that a 1-h peptide incubation was sufficient to block HPV-Lucia infection (Fig. 5C). Overall, these studies suggest that the L2N lipopeptide acts on the early step of HPV entry.

10.1128/mBio.01834-19.6 FIG S6 L2N lipopeptide does not bind to HPV virion particles to block infection. (A) CHO-K1 cells were infected with EdU-labeled HPV in presence of 1 μM FITC-m13-46st peptide for 4 h. Cells were fixed, followed by HPV DNA staining through a click reaction by using a Click-iT Plus EdU Alexa 555 imaging kit. Bars, 5 μm. Download FIG S6, JPG file, 2.8 MB. Copyright © 2019 Yan et al. 2019 Yan et al. This content is distributed under the terms of the Creative Commons Attribution 4.0 International license.

To further dissect how the L2N lipopeptide interfered with HPV entry, we tested whether furin cleavage, internalization, and JWW-1 epitope exposure of HPV were affected by peptide treatment in either CHO-K1 cells or HeLa cells. Furin cleavage was analyzed by infection of HPV PsV carrying Propionibacterium shermanii transcarboxylase domain (PSTCD)-fused L2. The N-terminal fusion of this small PSTCD (9 kDa) does not affect viral infectivity but can generate a detectable molecular weight shift upon furin cleavage (39). Cells were infected with HPV PsV carrying PSTCD-fused L2 in the presence of L2N lipopeptide or other entry inhibitors for 6 h and then lysed for immunoblot analysis of L2 cleavage. Our data showed that furin cleavage was inhibited by a furin inhibitor, dRVRK, but not by 13-46st lipopeptide, bafilomycin A1 (BafA1), or γ-secretase inhibitor XXI (XXI) (Fig. 5D). Internalization of viral particles was analyzed by detecting intracellular L1 and L2 at 2 h postinfection. We found that similar levels of intracellular L1 and L2 were detected upon treatment with 13-46st lipopeptide or BafA1, suggesting that viral internalization was not affected by 13-46st lipopeptide treatment (Fig. 5E). Furin cleavage of HPV has been shown to trigger the exposure of the RG1/JWW-1 epitope (20). However, treatment with the FITC-m13-46st lipopeptide, which is not recognized by JWW-1 antibody, showed no effect on RG1/JWW-1 epitope exposure (Fig. 5F). These results collectively indicate that L2N lipopeptide treatment does not affect HPV furin cleavage, internalization, and JWW-1 epitope exposure.

### The L2N lipopeptide blocks TGN trafficking of HPV, leading to rapid virion degradation.

**FIG 6:**
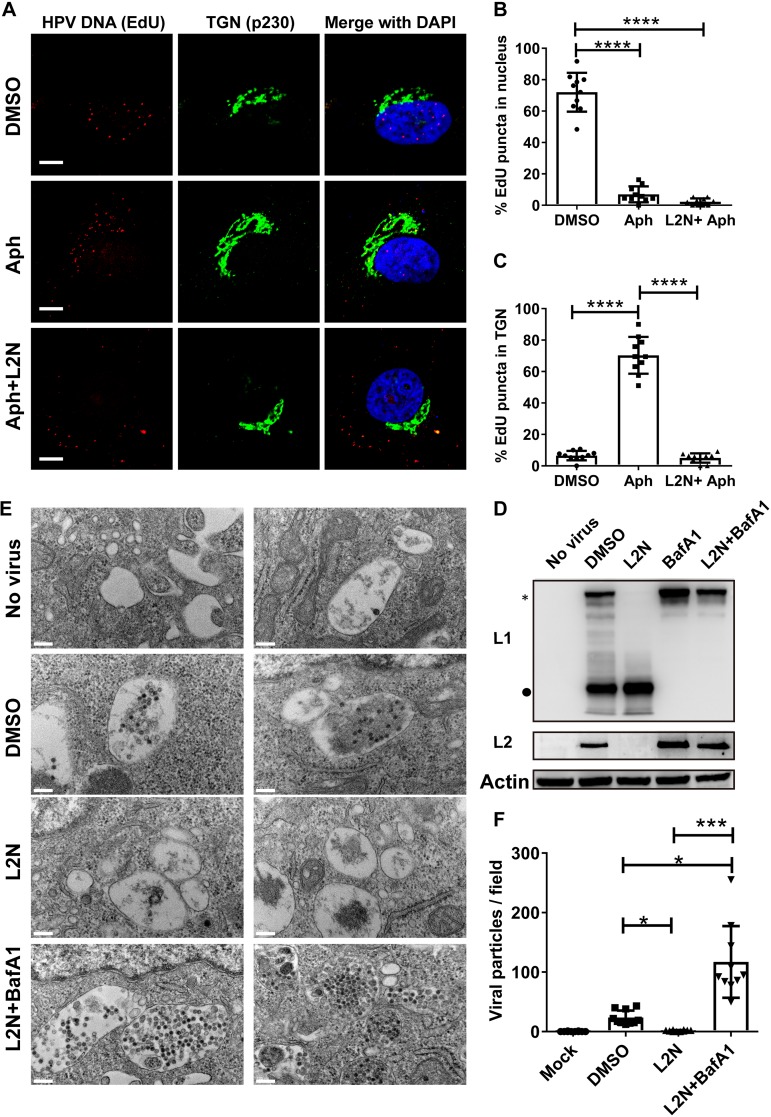
The L2N lipopeptide blocks TGN trafficking of HPV, leading to rapid virion degradation. (A) HPV retrograde trafficking to the TGN is blocked by L2N lipopeptide. HeLa cells were infected with EdU-labeled HPV (MOI of 50) and treated with DMSO, 3 μM aphidicolin (Aph), or 3 μM aphidicolin plus 500 nM 13-46st peptide (Aph+L2N). Cells were washed at 6 hpi and cultured for an additional 24 h with the indicated chemicals. HPV vDNA was visualized by a click reaction via Alexa 555 azide (red). Localization of the TGN (green) was analyzed by immunostaining with anti-P230 antibody. Bars, 5 μm. (B and C) Cellular distribution of EdU puncta. The ratios of EdU puncta in the nucleus (B) or in the TGN (C) were quantified based on representative images under each condition. *P* values were determined by ordinary one-way ANOVA with Dunnett’s multiple-comparison test (*n* = 10) ****, *P* < 0.0001. (D) CHO-K1 cells were infected with HPV-GFP (MOI of 50) and treated with 500 nM L2N lipopeptide 13-46st, 100 nM BafA1, or both for 24 h. Intracellular L1 and L2 levels were determined by immunoblotting with MD2H11 and JWW-1, respectively. *, full-length L1 (50 kDa); ●, 22-kDa L1 degradation fragment. (E and F) Ultrastructural analysis of intracellular HPV particles. (E) HeLa cells were infected with HPV at an MOI of 500 (about 10,000 particles/cell) for 16 h with or without treatment with 100 nM BafA1 and 500 nM 13-46st. Cells were processed for thin-section electron microscopy according to standard procedures, as described in Materials and Methods. Bars,100 nm. (F) Quantification of viral particles in representative images under each condition. One-way ANOVA (Kruskal-Wallis test) and Dunn’s multiple-comparison test were used for statistical analysis (*n* = 10). ***, *P* < 0.001; *, *P* < 0.05.

Next, we tested whether the L2N lipopeptide affected the retrograde trafficking of HPV to the TGN compartment. The accumulation of HPV L2/viral DNA (vDNA) in the TGN can be detected in the presence of the G_1_/S-phase transition inhibitor aphidicolin (Aph), as the translocation of HPV vDNA (viral DNA) from the TGN to the nucleus requires the onset of mitosis (32). HeLa cells were infected with EdU-labeled HPV and treated with or without 13-46st lipopeptide. Click reactions were conducted to analyze the subcellular localization of vDNA. Our data showed that HPV vDNAs were readily detected in the nucleus without Aph treatment, while they accumulated at the TGN upon Aph treatment (Fig. 6A to C). Interestingly, the overall HPV vDNA-positive signals were significantly reduced upon L2N lipopeptide treatment and no longer colocalized with the TGN P230 marker.

We hypothesized that HPV failed to reach the TGN in the presence of L2N lipopeptide and ultimately underwent rapid lysosomal degradation. To test this, HPV-GFP-infected cells were treated with and without 13-46st lipopeptide and/or BafA1 for 24 h. Treatment with 13-46st lipopeptide led to a drastic decrease of intracellular full-length L1 and L2 levels along with a considerable increase of the 20-kDa L1 degraded product. In contrast, BafA1 treatment prevented L1 and L2 degradation regardless of 13-46st lipopeptide treatment (Fig. 6D). Electron microscopy analysis showed that the numbers of intracellular HPV particles were significantly reduced upon 13-46st lipopeptide treatment at 16 hpi. Indeed, we observed endosomal accumulation of HPV particles when cells were treated with BafA1 and 13-46st lipopeptide (Fig. 6E and F). These results suggest that the L2N lipopeptide blocks HPV infection by preventing viral endosomal escape to the TGN, resulting in rapid viral degradation.

### L2N peptide-mediated inhibition of various papillomavirus infections.

**FIG 7:**
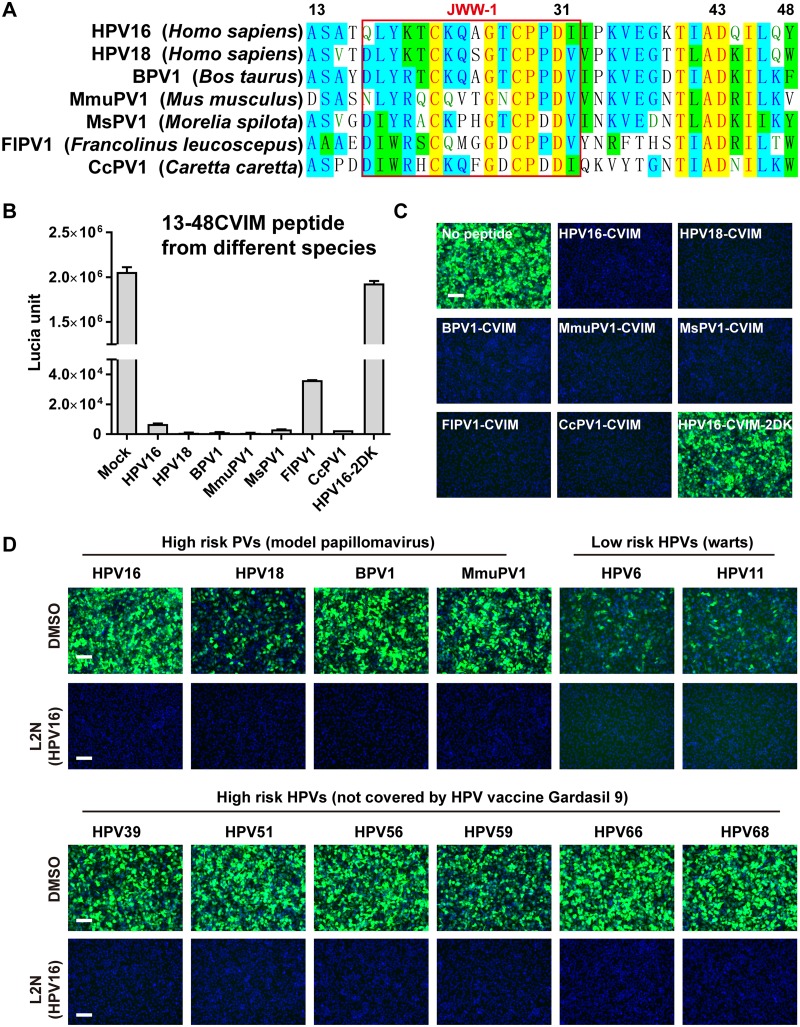
L2N peptide-mediated inhibition of various papillomavirus infections. (A) Sequence alignment of L2 N-terminal aa 13 to 48 (based on HPV16) from various papillomaviruses. (B and C) HeLa cells were infected with HPV16-GFP/Lucia and treated with L2N-13-48CVIM lipopeptides derived from various papillomaviruses (∼500 nM). The HPV16-2DK-CVIM (D_31_K and D_43_K) lipopeptide was included as a negative control. Lucia activity (B) and GFP intensity (C) were analyzed at 36 hpi (*n* = 2). Bar, 200 μm. (D) HeLa cells were infected with the indicated papillomaviruses (MOI of 1 to 5) and treated with or without 100 nM synthetic L2N lipopeptide of the HPV16 L2 sequence (13-46st). GFP intensity was examined at 36 hpi. Bars, 200 μm.

Sequence alignment shows that the N-terminal region spanning aa 13 to 48 of L2 contains highly conserved residues among papillomavirus from mammals, snakes, turtles, and birds (Fig. 7A), suggesting that the L2N peptide has an evolutionarily conserved function. To confirm this, we generated CAAX-mediated L2N lipopeptides derived from HPV16, HPV18, MmuPV1, bovine papillomavirus 1 (BPV1), snake Morelia spilota papillomavirus 1 (MsPV1), bird Francolinus leucoscepus papillomavirus 1 (FlPV1), or turtle Caretta caretta papillomavirus 1 (CcPV1). Our data demonstrated that the 13-48CVIM peptides derived from various PVs effectively blocked HPV16 infection, whereas the 13-48CVIM-D_31_,_43_K (2DK) mutant peptide showed no inhibition (Fig. 7B and C). The 13-48CVIM lipopeptide derived from FlPV1 showed the lowest inhibition activity among all peptides, yet it still blocked 98% of HPV16 infection at a concentration of 500 nM. Furthermore, the synthetic 13-46st lipopeptide derived from HPV16 was able to block the entry of various papillomaviruses, including high-risk PVs (HPV16, HPV18, MmuPV1, and BPV1), low-risk PVs (HPV6 and HPV11), and other high-risk HPVs (HPV39, HPV51, HPV56, HPV59, HPV66, and HPV68) that are not covered by the HPV vaccine Gardasil 9 (Fig. 7D). Together, these results suggested that the anti-HPV activity of the L2N peptide is mediated by the highly conserved L2 N-terminal sequence of various papillomaviruses.

## DISCUSSION

Several studies have shown that the N-terminal region of L2 is exposed after binding to the extracellular matrix and cell surface, an event that is dependent on conformational changes and furin cleavage (18, 20, 39, 44). The N-terminal region of L2 contains a broadly neutralizing epitope, RG1/JWW-1, which is ideal for the development of L2 based vaccines (19, 21, 45–48). Yet how the N-terminal region of L2 contributes to the early steps of HPV entry remains elusive. Sequence alignment indicates that the N-terminal aa 13 to 75 of L2 are highly conserved among 118 different HPVs (see Fig. S1 in the supplemental material). A previous study of the RG1 region (40) has shown that most single-alanine substitutions in this region except two cysteines for disulfide bonding have no effect on HPV infectivity. Another study, however, demonstrated the importance of glycine residues of the GXXXG motif for HPV infection (34). Here, we show that this evolutionarily conserved sequence between the furin cleavage sequence and a putative TM domain of L2, termed L2N, was a major contributor to HPV infectivity. Specifically, the two conserved aspartic acids D_31_ and D_43_ and their adjacent sequences were critical for HPV infection. Therefore, we seek to identify the role of this evolutionarily conserved sequence of L2 for HPV infection.

In this study, we used four approaches to achieve the surface display of the L2 N-terminal region: (i) secretion-carrier fusion, (ii) IL-2Rα TM replacement, (iii) CAAX motif-mediated isoprenylation, and (iv) synthetic stearoylation. Strikingly, these four approaches demonstrated that the surface display of the L2 N-terminal region rendered cells resistant to HPV infection and that the N-terminal aa 13 to 46 of L2 and the membrane-anchoring ability were essential for the inhibitory activity. The synthetic 13-46st lipopeptide functioned as a specific cross-type and potent HPV entry inhibitor, which effectively prevented infection by various types of HPV at a picomolar level (IC_50_ of ∼200 pM for HPV16). We also showed that the furin cleavage and TM-mediated or lipidation-mediated membrane-anchoring abilities of the L2N peptide were indispensable for the inhibition of HPV infection. Interestingly, Yang et al. (49) reported that both the L2 aa 13 to 31-GFP fusion protein of HPV16 and the bovine papillomavirus (BPV) L2 peptide spanning aa 1 to 88 have cell surface binding ability. Specifically, the BPV L2 peptide spanning aa 1 to 88 contains intact furin cleavage sites and N-terminal residues, which enables the inhibition of BPV infection at a concentration of 10 μg/ml (∼10.9 μM) (49). The inhibitory effect of the BPV peptide spanning aa 1 to 88 was later proposed to be due to its interaction with β-actin, which directs the cytoplasm transport of BPV to the perinuclear region (50). It is likely that the HPV L2N lipopeptide may utilize a molecular mechanism different from the one utilized by the BPV peptide spanning aa 1 to 88 to inhibit viral infection. Moreover, the proposed mechanism of β-actin-mediated intracellular trafficking remains controversial, since the L2 N terminus has been characterized as being located within the lumen side throughout the viral entry process (41, 51). Thus, future study is warranted for investigating how these peptides block papillomavirus infection.

Our results indicated that the L2N lipopeptide acted at the early entry step of HPV infection. Specifically, the L2N lipopeptide appeared to prevent viral endosomal escape, as HPV particles did not reach the TGN and instead underwent rapid degradation, which could be blocked by the endosomal acidification inhibitor BafA1. However, the specific mode of action of L2N lipopeptide needs to be further studied. Considering the strict requirement for accurate N-terminal furin cleavage of L2N lipopeptide for its activity, we assume that an interaction between the viral L2 N-terminal region and an unknown host membrane protein may be a critical early step of HPV entry. While it has been well studied that L2 furin cleavage is essential for HPV infection, it remains elusive why this cleavage is critical (17, 18). Our results suggest that furin cleavage may result in exposing the motif or sequence in the L2 N-terminal region that may initially be masked by the N-terminal positively charged residues for nuclear localization and the furin cleavage sequence (52–54). The critical C_20_-C_28_ disulfide bonding may make these positively charged residues physically close to the two negatively charged D_31_ and D_43_ residues that were shown to be required for HPV infection (Fig. 1B). It is also tempting to speculate that the sequential conformational change induced by furin cleavage and RG1/JWW-1 neutralizing epitope exposure may be the strategy used by HPV to achieve spatiotemporal control of the critical interaction between the L2 N-terminal region and an unknown host membrane protein. HPV neutralization antibodies that target the RG1/JWW-1 epitope of L2 may block the interaction with the host membrane protein on the cell surface and/or in the endosomal compartment. Further detailed study is needed to provide evidence for or against this hypothesis.

Intriguingly, the feature of the HPV L2N lipopeptide is very similar to that of the hepatitis B virus (HBV) preS1 lipopeptide that binds the HBV receptor sodium taurocholate cotransporting polypeptide (NTCP) and blocks HBV entry (55, 56). In both cases, lipopeptides derived from the N-terminal conserved region of viral minor proteins can efficiently block viral entry at low concentrations. Interestingly, the HBV preS1 lipopeptide has a type II TM topology through N-terminal lipidation, while the HPV L2N lipopeptide has a type I TM topology through C-terminal lipidation. As the HBV preS1 lipopeptide blocks viral entry by binding its entry receptor, it would be interesting to test whether the HPV L2N lipopeptide also targets an unknown entry receptor to block viral infection. It remains unclear whether the potential receptor is expressed on the cell surface or in the endosomal compartment or is dynamically distributed in both places. As we observed a rapid uptake of FITC-m13-46st lipopeptide, we speculate that this peptide may bind a cell surface protein to trigger its endocytosis but actually functions in the endosomal compartment. Future studies will be focused on the identification of the host target(s) of the L2N lipopeptide, which ultimately will reveal molecular insight into the early step of HPV entry.

In summary, the high sequence conservation of the L2 N-terminal region and the cross-type inhibitory activity of the L2N lipopeptide strongly indicate that the N-terminal region of L2 spanning aa 13 to 46 plays an important role in HPV entry, and the L2N lipopeptide may interact with a host membrane protein(s), ultimately blocking HPV infection. Thus, the L2N lipopeptide can be employed as a useful tool to study HPV entry and target identification. Furthermore, the potent, specific, and cross-type features of the L2N lipopeptide also open an opportunity to develop a peptide-mediated prevention strategy against HPV infection.

## MATERIALS AND METHODS

### Cell lines, antibodies, and other reagents.

Cell lines, antibodies, chemical inhibitors, and commercial kits used in this study are listed in Table S3 in the supplemental material.

10.1128/mBio.01834-19.9 TABLE S3 Key resources. Shown is a list of cells, antibodies, inhibitors, and other reagents used in this study. Download Table S3, PDF file, 0.1 MB. Copyright © 2019 Yan et al. 2019 Yan et al. This content is distributed under the terms of the Creative Commons Attribution 4.0 International license.

### Cell culture.

All cells listed in Table S3 were cultured at 37°C with 5% CO_2_ in the indicated growth medium with regular passage every 2 to 3 days. Stable cell lines were maintained in culture medium with an appropriate concentration of puromycin (10 μg/ml for CHO-K1 cells and pgsA-745 cells and 2 μg/ml for other cell types). Primary human epidermal keratinocytes were cultured on collagen-coated plates (catalog number R011K; Gibco) and maintained in EpiLife medium (catalog number M-EPI-500-CA; Gibco) supplemented with EpiLife defined growth supplement (EDGS) (catalog number S-012-5; Gibco).

### Plasmid constructions.

Plasmids for transient expression of proteins in mammalian cells were constructed based on pZeo1 (made in-house from p16L1L2 [Addgene]) and pZeo5 (made in-house, modified from pFUSEN [InvivoGen]) vectors with the EF1α promoter and a zeocin antibiotic marker. Plasmids for lentiviral transduction were generated based on the pCDH-CMV-MCS-EF1-Puro vector. Plasmids expressing different L1/L2 proteins for HPV pseudovirus (PsV) production were purchased from Addgene (John Schiller’s lab and Richard Roden’s lab). p16L1L2-PSTCD-3F expressing WT L1 and PSTCD-L2-3×FLAG was constructed from p16L1L2 by inserting the PSTCD sequence (N terminus) and 3×FLAG tag (C terminus) into the L2 coding sequence. pZeo1-L2 expressing WT L2 was generated by inserting the HPV16 L2 sequence into pZeo1. L2 N-terminal region mutants were generated from pZeo1-L2 through reverse PCR. Reporter plasmid pCI-neoGFP used for HPV PsV encapsidation was purchased from Addgene, and pCI-Lucia was generated from pCI-neoGFP by replacing the GFP sequence with the Lucia sequence that was amplified from pNiFty3-Lucia (pnf3-lc1; InvivoGen). To generate plasmids for surface display of the L2 N-terminal region, various lengths of HPV16 L2 N-terminal sequences (from aa 6 to different C-terminal ends) were fused to the C terminus of the Lucia sequence or Lucia signal peptide sequence (MEIKVLFALICIAVAEA) and cloned into pCDH-CMV-MCS-EF1-Puro. In some plasmids, the interleukin-2 receptor subunit alpha (IL-2Rα) TM sequence (VAVAGCVFLLISVLLLSGL) was fused after aa 48 of the L2 N-terminal region, followed by a Strep-tag sequence (WSHPQFEK) or 3×FLAG sequence at the C terminus. To generate constructs expressing L2N-CVIM peptide, L2 N-terminal sequences (from aa 6 to different C-terminal ends) were fused with an N-terminal Twin-Strep-tag (tSA) sequence (WSHPQFEKGGGSGGGSGGSAWSHPQFEK) and a C-terminal CVIM sequence. To generate the pCDH-puro-dFur713 plasmid for the establishment of the 293TdF cell line, the sequence of the furin ectodomain (aa 1 to 713) was amplified from FURIN-pIRESpuro2 (a generous gift from Richard Roden’s lab) and cloned into pCDH-CMV-MCS-EF1-Puro. Mutations and deletions within L2 N-terminal sequences were constructed by reverse PCR. In most plasmids, short GlyThr or GlySer linkers were inserted between different coding sequences. Additional information on these plasmids is listed in Tables S1 and S2.

### Production of HPV PsV.

pCI-neoGFP and/or pCI-Lucia was used as the reporter plasmid for HPV PsV encapsidation. PsVs of HPV16 or other papillomaviruses were produced by cotransfecting 293TT cells with L1/L2-expressing plasmids (such as p16L1L2 or p16sheLL) and reporter plasmids (pCI-neoGFP and/or pCI-Lucia) at a 1:1 ratio. PsVs were maturated and purified based on previously described protocols (57, 58), with an improved maturation method (http://home.ccr.cancer.gov/lco/ImprovedMaturation.htm). Specifically, cells were lysed at 48 h posttransfection by using 0.5% Triton X-100–phosphate-buffered saline (PBS) with 25 mM (NH_4_)_2_SO_4_ (pH 9), 1× protease inhibitor cocktail (catalog number 11697498001; Sigma), and 1× RNase (catalog number LSKPMRN30; EMD Millipore) and kept at 37°C overnight for maturation. The mature PsV from the lysate was further purified by ultracentrifugation on a discontinuous Optiprep (catalog number D1556; Sigma-Aldrich) density gradient (27% to 33% to 39%). HPV PsVs carrying L2 mutants were produced by cotransfection of pZeo1-L1 and pZeo1-L2 mutants at a 1:3 ratio. The purity and yield of purified PsVs were evaluated by Coomassie blue staining compared with a bovine serum albumin (BSA) standard. PsV particle numbers of the PsV stock were determined based on a previously described protocol (58). Specifically, 6 μl of a virus-containing solution was mixed with 2 μl 4× master mix (5% proteinase K solution, 2.5 mM EDTA, 0.5% SDS) and incubated at 56°C for 30 min. The treated product was loaded onto an agarose gel together with serial dilutions of pCI-neoGFP plasmids. The infectious titer (multiplicity of infection [MOI]) of WT PsV was determined by immunofluorescence or fluorescence-activated cell sorter (FACS) analysis of GFP expression in CHO-K1 cells at 48 hpi. The infectivity/particle ratio of PsV preparations is about 1:200 in our hands. EdU labeled HPV PsV was produced by culturing transfected 293TT cells with growth medium supplemented with 100 μM EdU at 6 h posttransfection (59).

### Furin production.

Lentivirus expressing the enzymatically active furin ectodomain (aa 1 to 713) was produced by cotransfection of 293T cells with pCDH-puro-dFur713 and helper plasmids. The 293TdF cell line stably expressing the furin ectodomain was established from 293T cells using lentiviral transduction and maintained in growth medium with 2 μg/ml puromycin. For furin production, 293TdF cells were cultured in Dulbecco’s modified Eagle’s medium (DMEM) with 10% fetal bovine serum (FBS) or Pro293 chemically defined serum-free medium (Pro293A-CDM; Lonza) for 2 days. The furin-containing supernatant was clarified by centrifugation at 4,000 rpm for 10 min, concentrated by using Amicon Ultra-15 centrifugal filter units (50-kDa cutoff; EMD Millipore) 50 times, and then stored at −20°C.

### JWW-1 antibody production.

The JWW-1 antibody is a human chimeric antibody with variable-region sequences derived from rat monoclonal antibody (mAb) WW-1, which is reactive with HPV16 L2 aa 18 to 32 (21). JWW-1 antibody was produced by transient transfection of 293T cells with pVItro-JWW-1 (catalog number 66748; Addgene) and purified by protein A/G-agarose. Specifically, growth medium of transfected 293T cells was replaced by Pro293A-CDM (Lonza). The antibody-containing supernatant was collected at 72 h posttransfection, followed by protein A/G-agarose (Thermo Scientific) purification according to the manufacturer’s instructions.

### Commercial peptide synthesis.

The synthetic peptides (Table 1) used in this study were synthesized by Biomatik Corporation. Peptide stocks were dissolved in dimethyl sulfoxide (DMSO) at a concentration of 1 mM and stored at −20°C.

### HPV infection assay.

Unless otherwise mentioned, 4 × 10^4^ freshly trypsinized cells seeded into a well of a 96-well plate were infected with WT or mutant HPV PsV at an MOI of 5 with exogenous furin (1/10 volume of 50× concentrated furin supernatant) at 37°C. At 16 hpi, infected cells were washed using fresh medium, and GFP intensity or Lucia activity was examined at 36 to 48 hpi. For the time course study in Fig. 5C, virus was added to the cells seeded for 12 h and incubated at 4°C for attachment and/or at 37°C for internalization. To visualize EdU-labeled vDNA, cells were seed onto coverslips in a 24-well plate at 1 × 10^5^ cells/well and infected with EdU-labeled HPV PsV at an MOI of 50.

### Lucia enzymatic assay.

To determine the bioluminescent activity of Lucia, 10 μl of the clarified supernatant from transfected or infected cells was mixed with 50 μl Quanti-Luc reagent (InvivoGen) in an opaque 96-well plate. The luminescence units were determined by using a FilterMax F5 multimode microplate reader (Molecular Devices).

### Cell surface display assay.

Stable cells expressing the L2 N-terminal region were established by lentiviral transduction and subsequently selected and maintained in growth medium with puromycin (10 μg/ml for CHO-K1 and pgsA-745 and 2 μg/ml for other cell types). Protein expression and HPV susceptibility of transduced cells were evaluated at 3 days postselection. The Lucia secretion signal peptide leads the fusion proteins to the secretion pathway, while the putative TM domain from L2 protein or the TM domain from IL-2Rα assists in tethering fusion proteins onto the plasma membrane. Upon viral infection with exogenous furin, N-terminal sequences upstream of the furin cleavage sequence along with the sequence (aa 8 to 12) itself will be removed by furin cleavage.

### L2N-CVIM lipopeptide production and purification.

To produce L2N-CVIM lipopeptides, 293T cells were transfected with pZeo5-tsa-furin-L2N-CVIM-based plasmids, which express L2N peptides with an N-terminal Twin-Strep-tag and a C-terminal CVIM motif. Specifically, 1 × 10^6^ transfected cells were lysed using 200 μl lysis buffer (PBS with 1% Triton X-100, 1× protease inhibitor cocktail, and 70 mU/ml of BioLock biotin blocking solution) at 48 h posttransfection. The L2N peptides carrying the Twin-Strep-tag were captured by using 1 μl settled MagStrep “type 3” XT beads (IBA Lifesciences) at 4°C for 1 h. After three washes with wash buffer (100 mM Tris-HCl [pH 8.0], 150 mM NaCl, and 1 mM EDTA), the peptides were released from the beads by furin cleavage by incubation with 10 to 20 μl of the 50× concentrated furin-containing supernatant at 37°C for 30 min. The concentration of the eluted peptide can be determined by immunoblotting using JWW-1 antibody with a synthetic L2N peptide of a known concentration. Generally, approximately 2 μg of L2N lipopeptide can be purified from 1 × 10^6^ transfected cells.

### Immunoblotting.

Cells were harvested in radioimmunoprecipitation assay (RIPA) buffer (20 mM Tris-Cl [pH 7.5], 150 mM NaCl, 1 mM EDTA, 1% Triton X-100, 0.5% sodium deoxycholate, 0.1% SDS) for immunoblot analysis. Proteins in the cell lysate were resolved in an SDS-PAGE gel, followed by a standard immunoblotting procedure. HPV16 L1 was detected by a mouse anti-HPV16 L1 mAb (MD2H11; Santa Cruz) at a 0.1-μg/ml concentration. For L2 N-terminal region mapping assays, WT and mutant HPV16 L2 were detected by a mouse anti-HPV16 L2 mAb (2JGmab#5; Santa Cruz) at 1 μg/ml. The JWW-1 mAb was used for immunoblot detection of L2N peptides and in L1 and L2 degradation assays at 1 μg/ml. Mouse anti-FLAG mAb (catalog number F1804; Sigma) was used for detection of the 3×FLAG tag at a 1:3,000 dilution. β-Actin was detected by anti-β-actin mAb (catalog number Sc-47779; Santa Cruz) at a 1:3,000 dilution. Goat anti-mouse and goat anti-human horseradish peroxidase (HRP)-conjugated secondary antibodies were used at a 1:10,000 dilution. The membrane was developed using the Amersham ECL chemiluminescent substrate (catalog number RPN2232; GE). Images were acquired using the ChemiDoc Touch imaging system (Bio-Rad).

### Immunofluorescence microscopy.

Cells for immunofluorescence analysis were fixed with 4% paraformaldehyde (PFA) for 10 min at room temperature (RT), washed twice with PBS, permeabilized with 0.25% Triton X-100 for 10 min at RT, and then blocked with PBS–3% BSA at 37°C for 1 h. JWW-1 mAb was used at 2 μg/ml to stain L2 proteins or L2N peptides. TGN marker anti-p230 (clone 15, catalog number 611280; BD) mAb was used at a 1:500 dilution. Cells were incubated with primary antibodies diluted in PBS–3% BSA at 37°C for 1 h and then washed three times with PBS and incubated with florescence-labeled secondary antibodies (1:1,000). To visualize EdU-labeled vDNA, the cells were washed and treated with Click-iT reaction buffer for 30 min at RT according to the instructions of Click-iT Plus EdU A555 imaging kit (catalog number C10638; Thermo Scientific). Coverslips were then mounted by using Prolong Antifade Diamond solution containing 4′,6-diamidino-2-phenylindole (DAPI) (catalog number P36971; Life Technologies) and imaged using a confocal microscope (Nikon Eclipse Ti).

### Flow cytometry analysis.

Surface expression of the L2 N-terminal region was analyzed with JWW-1 antibody. Specifically, live cells without fixation were detached by using 5 mM EDTA–PBS and stained with JWW-1 antibody at 20 μg/ml for 30 min on ice, followed by fluorescence-conjugated secondary antibody (1:200) staining for 30 min on ice. The stained cells were analyzed with an Attune NxT flow cytometer (Thermo Fisher). All FACS data were analyzed using FlowJo software.

### L2N-FITC peptide binding and uptake assay.

Fluorescein isothiocyanate (FITC)-labeled L2N lipopeptide (FITC-m13-46st) was used for tracing the binding and localization of L2N lipopeptide in the cells. MmuPV1 L2N aa 13 to 46 are not reactive with JWW-1 antibody, rendering it feasible to costain HPV16 virions with JWW-1. To visualize L2N lipopeptide binding, cells at 90% confluence were incubated with 1 μM FITC-m13-46st peptide diluted in DMEM for the indicated times. Cells were washed with PBS three times and then fixed with 4% PFA for 10 min at room temperature. Nuclei were stained with DAPI.

### Immunoblot detection of intracellular L1 and L2.

Cells were seed in a 48-well plate at 5 × 10^4^ cells/well, infected with HPV PsV at an MOI of 50, and treated with or without chemicals. To detect intracellular HPV L1 and L2, the infected cells were washed with culture medium twice, incubated with high-pH phosphate buffer (PBS; pH 10.7) at RT for 2.5 min, and subsequently incubated with 0.5% trypsin at 37°C for 30 min to remove surface-bound viruses (35). Next, cells were lysed by using RIPA buffer for immunoblot detection of intracellular L1 and L2 levels by MD2H11 and JWW-1, respectively.

### Electron microscopy.

A total of 1 × 10^6^ HeLa cells cultured in a 6-well plate were infected with HPV-GFP at an MOI of 500 (about 10,000 particles per cell) in the presence of the indicated chemicals for 16 h. Samples were treated using a modified protocol as previously described (60). Specifically, cells were fixed with one-half-strength Karnovsky’s fixative (2.5% glutaraldehyde and 2% PFA in 0.1 M phosphate buffer, for 30 min at RT) and rinsed with 0.1 M cacodylate buffer, followed by a 2-h incubation in 2% aqueous OsO_4_–0.2 M cacodylate buffer in a 1:1 solution on ice. Cells rinsed with 0.1 M cacodylate buffer were subsequently stained *en bloc* with uranyl acetate overnight at 4°C. The cells were dehydrated with a graded series of ethanol and embedded in fresh Eponate resin for 24 h. Ultrathin sections (∼70 nm) of samples were placed onto grids. The sections were examined with a JEM-2100 transmission electron microscope (JEOL Ltd.).

### HPV L2 sequence alignment and analysis.

L2 sequences from different HPVs or animal PVs were retrieved from the NCBI or UniProt database. The sequence alignment and similarity analysis were conducted by using Vector NTI software. The residue frequency map of the L2 N-terminal region was generated by using WebLogo online software (https://weblogo.berkeley.edu/logo.cgi).

### Statistical analysis.

All experiments were repeated at least twice, with similar results. Unless otherwise mentioned, results are represented as means ± standard deviations determined using GraphPad Prism 7. One-way analysis of variance (ANOVA) with a software-recommended comparison test was used to assess statistical significance. *P* values are indicated in the figure legends.
