# Supplementary figures and images for: Efficient Inhibition of Human Papillomavirus Infection by L2 Minor Capsid-Derived Lipopeptide
Source: mBio. 2019 Aug 6;10(4):e01834-19. doi: 10.1128/mBio.01834-19 (PMC6686047; doi:10.1128/mBio.01834-19)

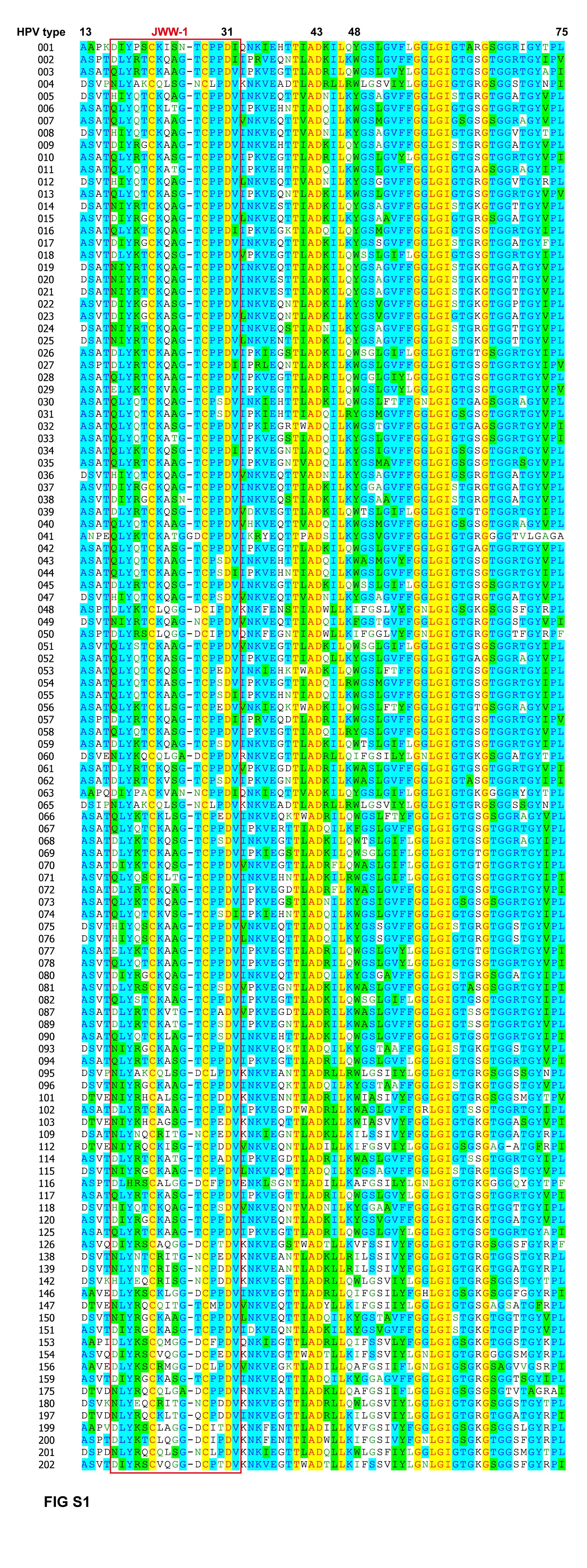

Supplement: FIG S1 [file mBio.01834-19-sf001.jpg]

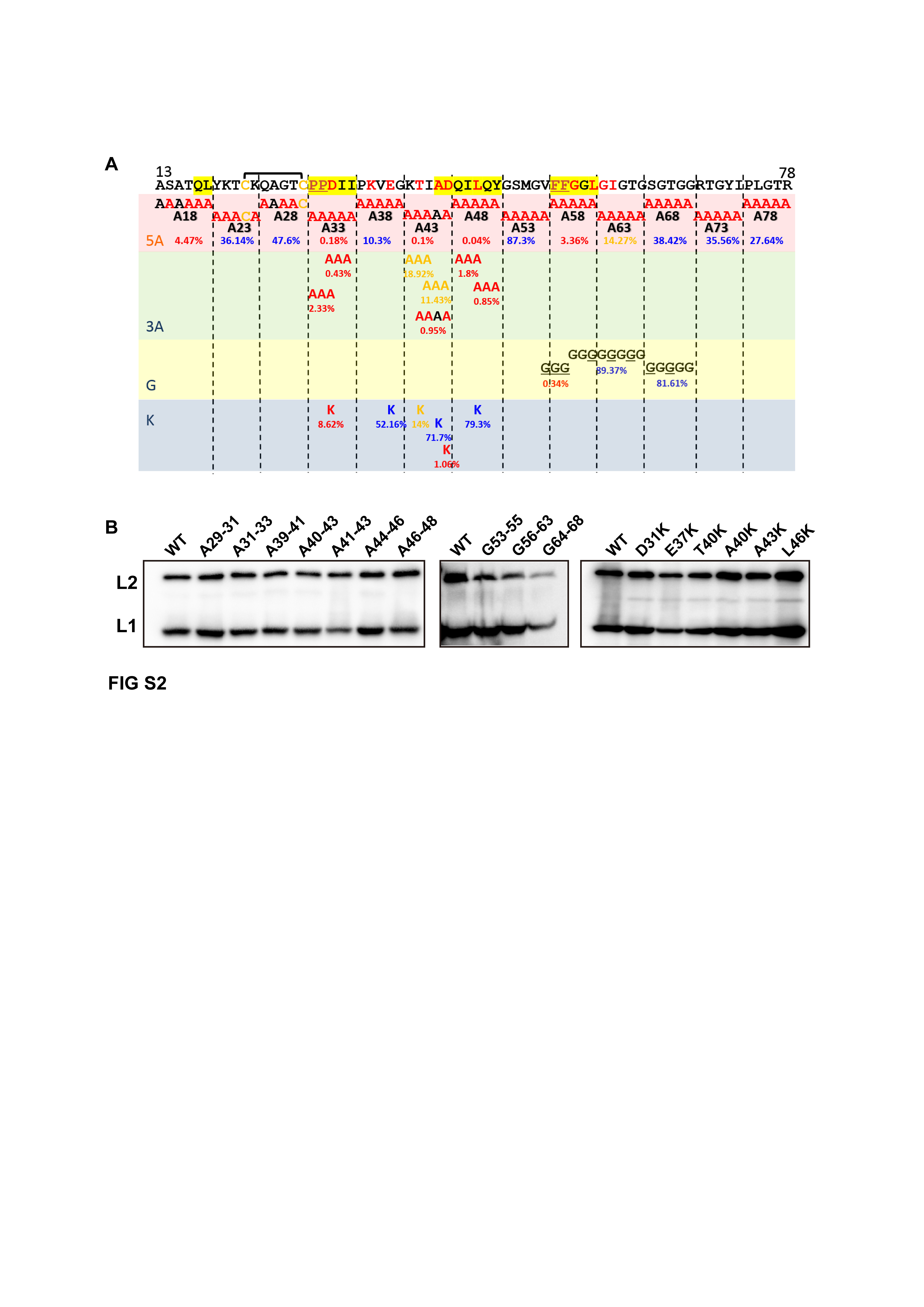

Supplement: FIG S2 [file mBio.01834-19-sf002.jpg]

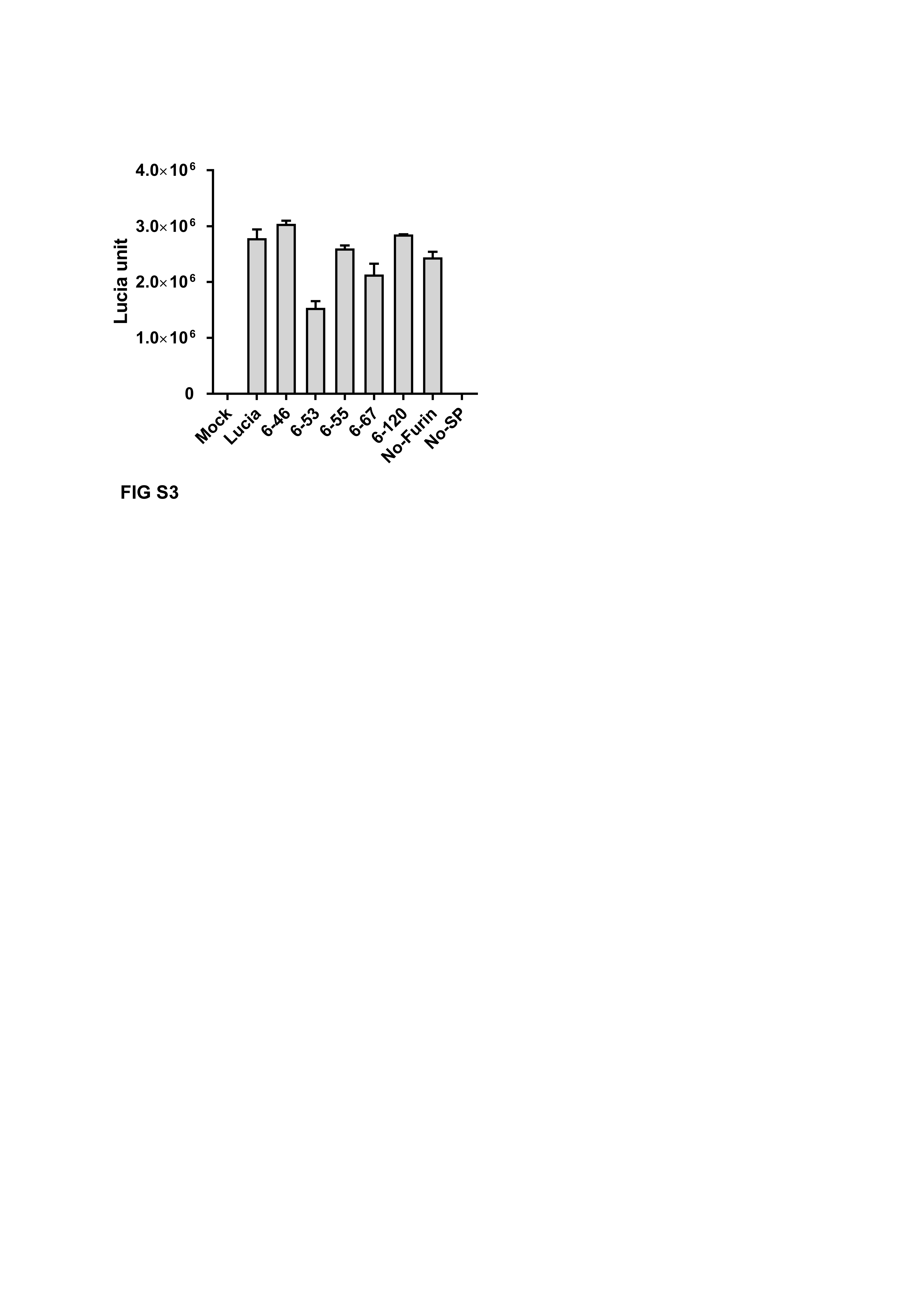

Supplement: FIG S3 [file mBio.01834-19-sf003.tif]

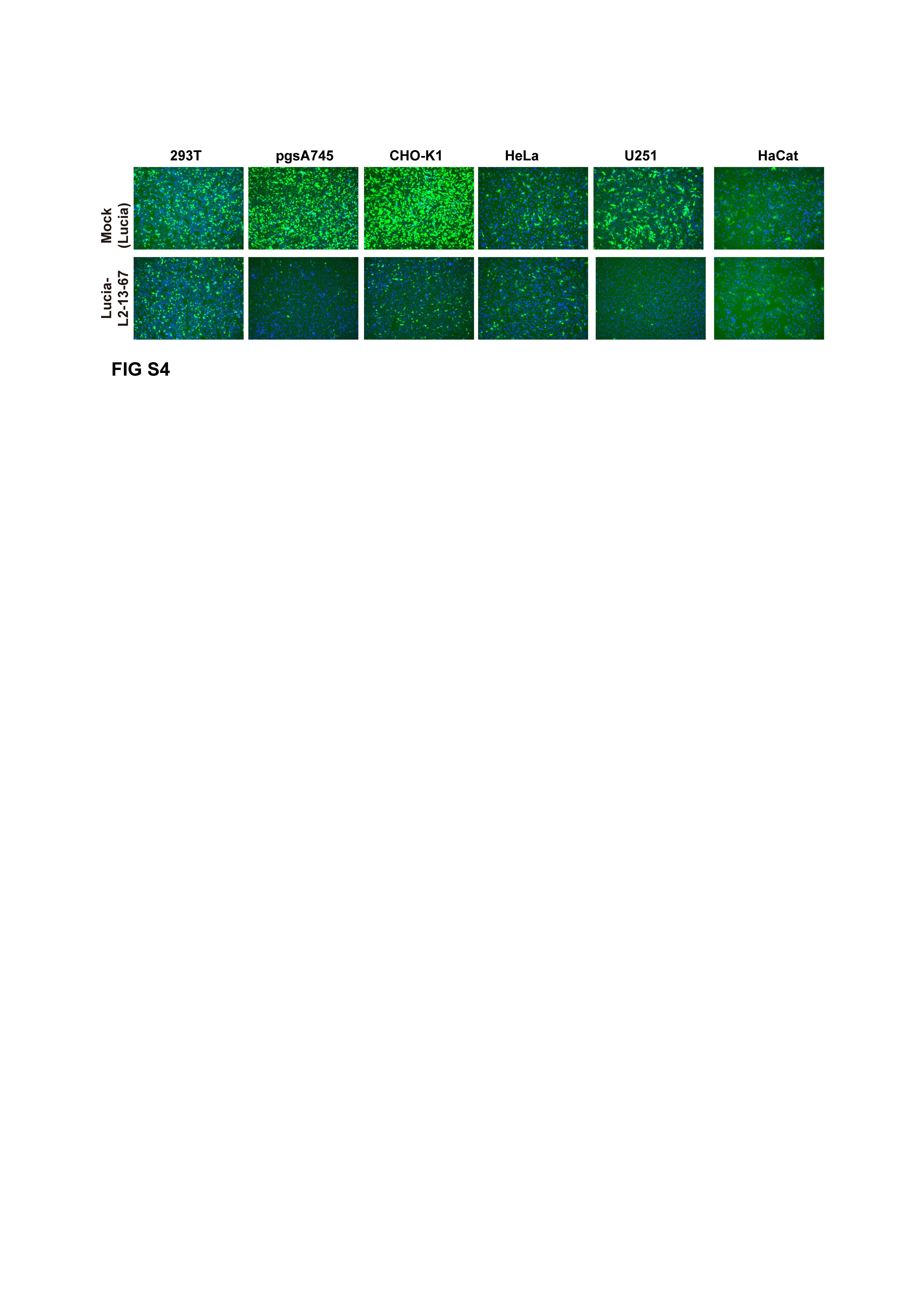

Supplement: FIG S4 [file mBio.01834-19-sf004.jpg]

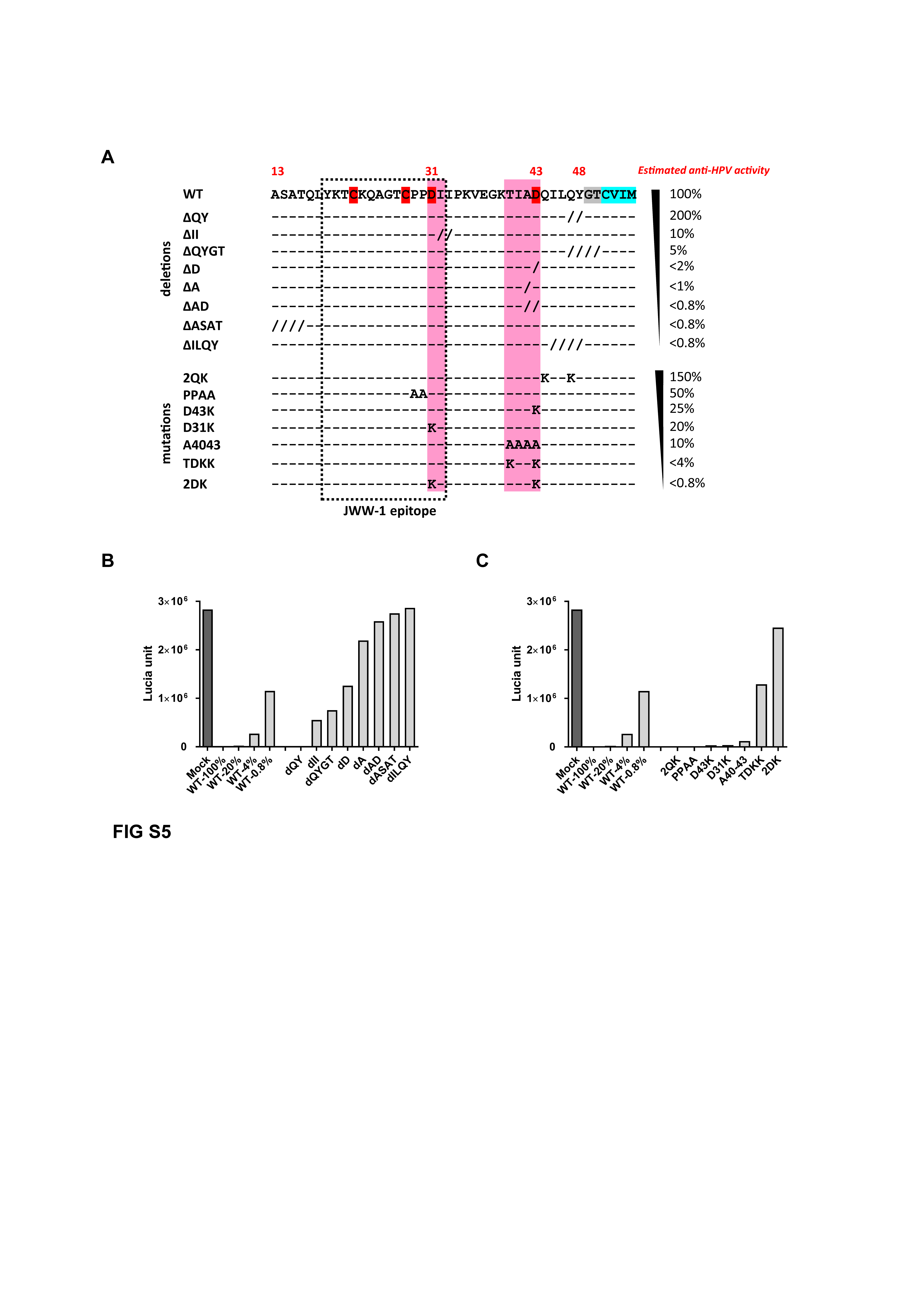

Supplement: FIG S5 [file mBio.01834-19-sf005.tif]

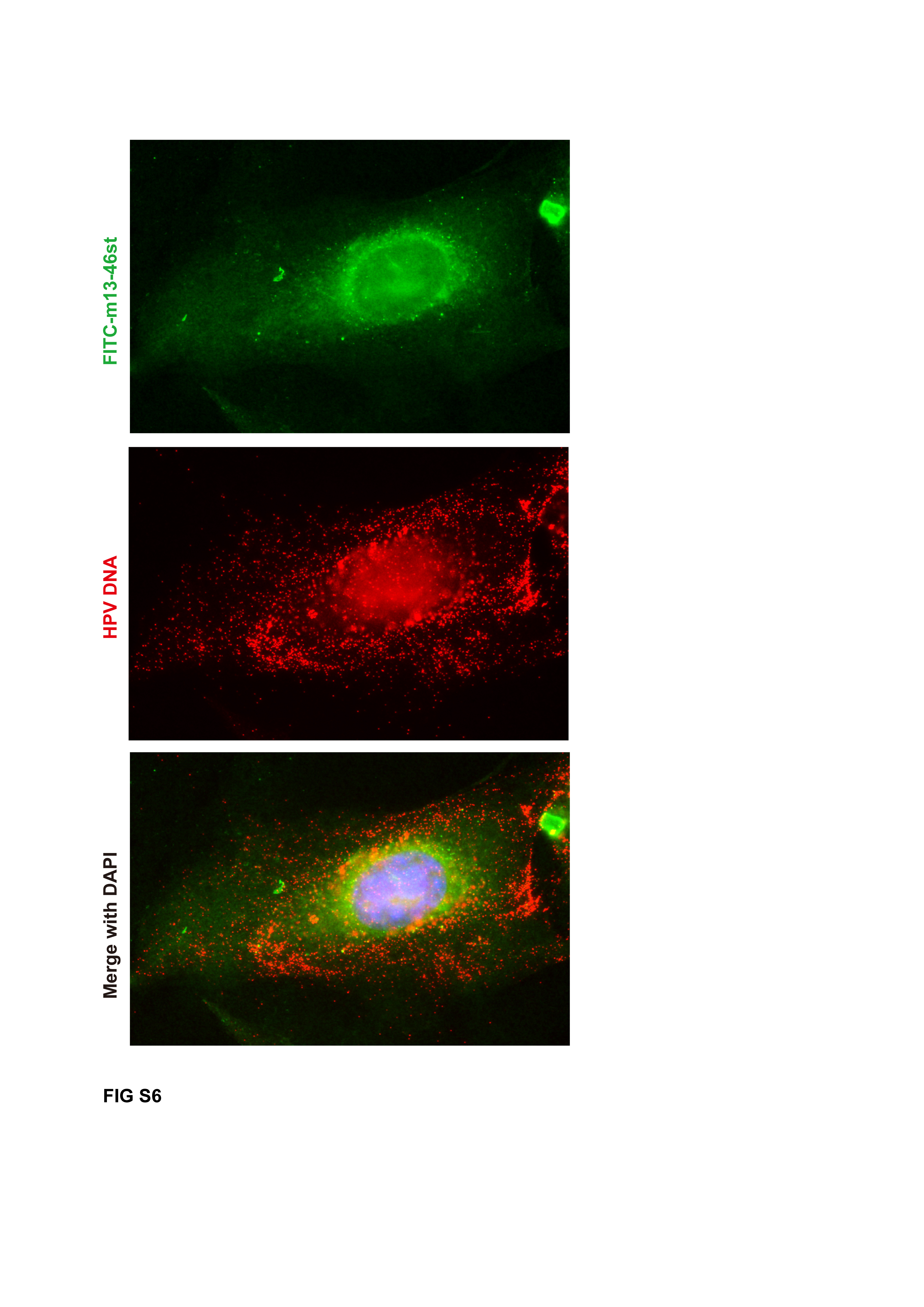

Supplement: FIG S6 [file mBio.01834-19-sf006.jpg]
