# Supplementary material for: Efficient Inhibition of Human Papillomavirus Infection by L2 Minor Capsid-Derived Lipopeptide
Source: mBio. 2019 Aug 6;10(4):e01834-19. doi: 10.1128/mBio.01834-19 (PMC6686047; doi:10.1128/mBio.01834-19)
Supplement: TABLE S1 [file mBio.01834-19-st001.pdf]

Table S1 List of plasmids to produce wild type and mutant HPV or other PVs

A

Plasmids for HPV PsV production	Source or reference	Identifiers	Additional information
pVItro-JWW-1	Addgene	66748	Production of JWW-1 mAb
pCDH-lenti-dFur713	This paper	NA	For establishing 293TdF cell line
pCI-Lucia	This paper	NA	production of HPV with lucia reproter
pCINeoEGFP	Addgene	46949	Production of HPV with EGFP reproter
p16L1L2	Addgene	45291	Production of HPV16 psv
p16L1L2-PSDTD-3F	This paper	NA	Production of HPV16 psv with L2-N PTCD fusion and C- 3FLAG fusion
p16SheLL	Addgene	37320	Production of HPV16 psv
pMusheLL	Addgene	47023	Production of MuPV psv
pSheLL	Addgene	37319	Production of BPVpsv
p18sheLL	Addgene	37321	Production of HPV18 psv
pVITRO-HPV6 L1L2	Addgene	52589	Production of HPV6 psv
pVITRO-HPV11 L1L2	Addgene	52590	Production of HPV11 psv
pVITRO-HPV39 L1L2	Addgene	52582	Production of HPV39 psv
pVITRO-HPV51 L1L2	Addgene	52583	Production of HPV51 psv
pVITRO-HPV56 L1L2	Addgene	52585	Production of HPV56 psv
pVITRO-HPV59 L1L2	Addgene	52586	Production of HPV59 psv
pVITRO-HPV66 L1L2	Addgene	52602	Production of HPV66 psv
pVITRO-HPV68 L1L2	Addgene	52587	Production of HPV68 psv

B

Plasmids for L2N fine mapping	Source or reference	Additional information	Relative HPV infectivity
**5A mapping**			
pZeo1-L1-WT	This paper	For HPV16 L1 only	0
pZeo1-L2-WT	This paper	For HPV16 L2	100%
pZeo1-L2-A13-16	This paper	For HPV16 L2 with 13-16 to Ala	91.80%
pZeo1-L2-A13-18	This paper	For HPV16 L2 with 13-18 to Ala	4.47%
pZeo1-L2-A19-23	This paper	For HPV16 L2 with 19-23 to Ala	36.14%
pZeo1-L2-A24-28	This paper	For HPV16 L2 with 24-28 to Ala	47.60%
pZeo1-L2-A29-33	This paper	For HPV16 L2 with 29-33 to Ala	0.18%
pZeo1-L2-A34-38	This paper	For HPV16 L2 with 34-38 to Ala	10.30%
pZeo1-L2-A39-43	This paper	For HPV16 L2 with 39-43 to Ala	0.10%
pZeo1-L2-A44-48	This paper	For HPV16 L2 with 44-48 to Ala	0.04%
pZeo1-L2-A49-53	This paper	For HPV16 L2 with 49-53 to Ala	87.30%
pZeo1-L2-A54-58	This paper	For HPV16 L2 with 54-58 to Ala	3.36%
pZeo1-L2-A59-63	This paper	For HPV16 L2 with 59-63 to Ala	14.27%
pZeo1-L2-A64-68	This paper	For HPV16 L2 with 64-68 to Ala	38.42%
pZeo1-L2-A69-73	This paper	For HPV16 L2 with 69-73 to Ala	35.56%
pZeo1-L2-A74-78	This paper	For HPV16 L2 with 74-78 to Ala	27.46%
**3A mapping**			
pZeo1-L2-A29-31	This paper	For HPV16 L2 with 29-31 to Ala	2.33%
pZeo1-L2-A31-33	This paper	For HPV16 L2 with 31-33 to Ala	0.43%
pZeo1-L2-A39-41	This paper	For HPV16 L2 with 29-41 to Ala	18.92%
pZeo1-L2-A40-43	This paper	For HPV16 L2 with 40-43 to Ala	0.95%
pZeo1-L2-A41-43	This paper	For HPV16 L2 with 41-43 to Ala	11.43%
pZeo1-L2-A44-46	This paper	For HPV16 L2 with 44-46 to Ala	1.80%
pZeo1-L2-A46-48	This paper	For HPV16 L2 with 46-48 to Ala	0.85%
**G mapping**			
pZeo1-L2-52-57G	This paper	For HPV16 L2 with 52-57 to Gly	0.34%
pZeo1-L2-56-63G	This paper	For HPV16 L2 with 56-63 to Gly	89.37%
pZeo1-L2-64-68G	This paper	For HPV16 L2 with 64-68 to Gly	81.61%
**K mapping**			
pZeo1-L2-D31K	This paper	For HPV16 L2 with D31K	8.62%
pZeo1-L2-E37K	This paper	For HPV16 L2 with E37K	52.16%
pZeo1-L2-T40K	This paper	For HPV16 L2 with T40K	14%
pZeo1-L2-A42K	This paper	For HPV16 L2 with A42K	71.70%
pZeo1-L2-D43K	This paper	For HPV16 L2 with D43K	1.06%
pZeo1-L2-L46K	This paper	For HPV16 L2 with L46K	79.30%
**A mapping**			
pZeo1-L2-C22A	This paper	For HPV16 L2 with C22A	3.17%
pZeo1-L2-D31A	This paper	For HPV16 L2 with D31A	34.03%
pZeo1-L2-D43A	This paper	For HPV16 L2 with D43A	43.25%
