# Supplementary material for: Efficient Inhibition of Human Papillomavirus Infection by L2 Minor Capsid-Derived Lipopeptide
Source: mBio. 2019 Aug 6;10(4):e01834-19. doi: 10.1128/mBio.01834-19 (PMC6686047; doi:10.1128/mBio.01834-19)
Supplement: TABLE S2 [file mBio.01834-19-st002.pdf]

Table S2 List of plasmids for expressing L2N fusion proteins or L2N-CVIM peptides

A

Motifs and affinity tags	Source or reference	Protein sequence	DNA sequence	Additional information
PSTCD	PMID: 27122588	EGEIPAPLAGTVSKILVKEGDTVKAGQTVLVLEAMKMETEINAPTDGKVE KVLVKERDAVQGGQGLIKIG	GAGGGCGAGATACCAGCCCCTCTGGCTGGT ACGGTCAGTAAAATTCTGGTTAAGGAGGGAG ATACCGTAAAGGCCGGGCAGACAGTCCTGG TGTTGGAAGCTATGAAGATGGAGACCGAGAT CAACGCCCCGACTGATGGAAAGGTGGAGAA GGTTCTGGTAAAGGAAAGGGACGCAGTGCA AGGTGGGCAGGGGCTCATAAAGATCGGC	Fused to N terminus of L2 in plasmid P16-L1L2--PSTCD-3F
IL2Rα TM	This paper	VAVAGCVFLLISVLLLSGL	GTGGCCGTGGCCGGCTGCGTTTTCCTGCTGA TCAGCGTGCTCCTTCTCAGCGGCCTG	replace HPV L2 TM
LuciaSP(Lsp)	This paper	MEIKVLFALICIAVAEA	ATGGAAATCAAGGTGCTGTTTGCCCTCATCT GTATTGCTGTTGCTGAGGCA	For surface expression
Streptag	This paper	WSHPQFEK	TGGAGCCACCCGCAGTTCGAAAAA	For Streptactin IP
Twin-Strep-tag (tSA)	This paper	WSHPQFEKGGGSGGGSGGSAWSHPQFEK	TGGAGCCACCCGCAGTTCGAAAAAGGTGGA GGTTCCGGCGGTGGATCGGGAGGTTCGGCG TGGTCCCATCCCCAGTTCGAGAAG	For Streptactin IP
3xFLAG tag (3F)	This paper	DYKDHDGDYKDHDIDYKDDDDK	GACTACAAGGACCACGACGGcGACTACAAG GACCACGACATCGACTACAAGGACGACGAC GACAAG	Used in constructs for IP and IB
CVIM motif	This paper	CVIM	TGTGTAATTATG	CAAX box for isoprenylation

B

Plasmids for L2N surface display	Source or reference	Additional information	Relative anti-HPV activity
pCDHp-Lucia-L2-13-46	This paper	For stable cell expressing Lucia-L2-13-46	-
pCDHp-Lucia-L2-13-53	This paper	For stable cell expressing Lucia-L2-13-53	+
pCDHp-Lucia-L2-13-55	This paper	For stable cell expressing Lucia-L2-13-55	++
pCDHp-Lucia-L2-13-67	This paper	For stable cell expressing Lucia-L2-13-67	++
pCDHp-Lucia-L2-13-120	This paper	For stable cell expressing Lucia-L2-13-120	-
pCDHp-Lucia-L2-13-55-no sp	This paper	For stable cell expressing Lucia-L2-13-55 without lucia SP (no SP)	-
pCDHp-Lucia-L2-13-55-R9,12K	This paper	For stable cell expressing Lucia-L2-13-55 with R9,12K	-
pCDHp-Lsp-13-48-TMtsa3F	This paper	For stable cell expressing Lsp-13-48-TMtsa3F	+++
pCDHp-Lsp-13-48-TMtsa3F-C20A	This paper	For stable cell expressing Lsp-13-48-TMtsa3F with C20A	-
pCDHp-Lsp-13-48-TMtsa3F-C28S	This paper	For stable cell expressing Lsp-13-48-TMtsa3F with C28S	-
pCDHp-Lsp-13-48-TMtsa3F-D31K	This paper	For stable cell expressing Lsp-13-48-TMtsa3F with D31K	-
pCDHp-Lsp-13-48-TMtsa3F-D43K	This paper	For stable cell expressing Lsp-13-48-TMtsa3F with D43K	-

C

Plasmids for L2N-CVIM lipopeptide production	Source or reference	Additional information	Relative anti-HPV activity
pzeo5-tSA-Furin-13-55GT-CVIM	This paper	For HPV16 L2N lipopeptide with L2N13-55	10%
pzeo5-tSA-Furin-13-48GT-CVIM	This paper	For HPV16 L2N lipopeptide with L2N13-48	100%
pzeo5-tSA-Furin-13-48GT-CVIM-No furin	This paper	For HPV16 L2N lipopeptide with L2N13-48 without R9K&R12K mutation	0%
pzeo5-tSA-Furin-13-48GT-SVIM	This paper	For HPV16 L2N lipopeptide with L2N13-48 with C to S mutation in CAAX	0%
pZeo5-tSA-Furin-13-46GT-CVIM	This paper	For HPV16 L2N lipopeptide with L2N13-46	200%
pZeo5-tSA-Furin-13-48GT-CVIM-d3233	This paper	For HPV16 L2N lipopeptide with L2N13-48 with deletion of II	10%
pZeo5-tSA-Furin-13-46-CVIM	This paper	For HPV16 L2N lipopeptide with L2N13-46 with deletion of QYGT	5%
pZeo5-tSA-Furin-13-48GT-CVIM-d43	This paper	For HPV16 L2N lipopeptide with L2N13-48 with deletion of D43	<2%
pZeo5-tSA-Furin-13-48GT-CVIM-d42	This paper	For HPV16 L2N lipopeptide with L2N13-48 with deletion of A42	<1%
pZeo5-tSA-Furin-13-48GT-CVIM-d42-43	This paper	For HPV16 L2N lipopeptide with L2N13-48 with deletion of AD	<0.8%
pZeo5-tSA-Furin-13-48GT-CVIM-d13-16	This paper	For HPV16 L2N lipopeptide with L2N13-48 with deletion of ASAT	<0.8%
pZeo5-tSA-Furin-13-44GT-CVIM	This paper	For HPV16 L2N lipopeptide with L2N13-48 with deletion of ILQY	<0.8%
pZeo5-tSA-Furin-13-48GT-CVIM-QQKK	This paper	For HPV16 L2N lipopeptide with L2N13-48 with double Q44K and Q47K	150%
pZeo5-tSA-Furin-13-48GT-CVIM-PPAA	This paper	For HPV16 L2N lipopeptide with L2N13-48 with double P32A and P33A	50%
pZeo5-tSA-Furin-13-48GT-CVIM-D43K	This paper	For HPV16 L2N lipopeptide with L2N13-48 with double D43K	25%
pZeo5-tSA-Furin-13-48GT-CVIM-D31K	This paper	For HPV16 L2N lipopeptide with L2N13-48 with double D31K	20%
pZeo5-tSA-Furin-13-48GT-CVIM-A40-43	This paper	For HPV16 L2N lipopeptide with L2N13-48 with A40-43	10%
pZeo5-tSA-Furin-13-48GT-CVIM-TDKK	This paper	For HPV16 L2N lipopeptide with L2N13-48 with T40K and D43K	<4%
pZeo5-tSA-Furin-13-48GT-CVIM-2DK	This paper	For HPV16 L2N lipopeptide with L2N13-48 with D31K and D43K	<0.8%
