# Supplementary material for: Efficient Inhibition of Human Papillomavirus Infection by L2 Minor Capsid-Derived Lipopeptide
Source: mBio. 2019 Aug 6;10(4):e01834-19. doi: 10.1128/mBio.01834-19 (PMC6686047; doi:10.1128/mBio.01834-19)
Supplement: TABLE S3 [file mBio.01834-19-st003.pdf]

Table S3 Key Resources Table

Reagent type (species) or resource	Designation	Source or reference	Identifiers	Additional information
cell line (homo sapiens)	293T	ATCC	CRL-3216	DMEM+10%FBS
cell line (homo sapiens)	293TT	Other	Gift from W. Martin Kast's lab, USC	DMEM+10%FBS+0.25 mg/ml hygromycin B
cell line (homo sapiens)	293TdF	This paper		DMEM+10%FBS+0.1 μg/ml puromycin
cell line (Cricetulus griseus)	CHO-K1	ATCC	CCL-61	DMEM/F12 +10%FBS
cell line (Cricetulus griseus)	pgsA745	ATCC	CRL-2242	DMEM/F12 +10%FBS
cell line (homo sapiens)	HeLa	ATCC	CCL-2	DMEM+10%FBS
cell line (homo sapiens)	HaCaT	Other	Gift from W. Martin Kast's lab, USC	DMEM+10%FBS
cell line (homo sapiens)	Huh7	Other	Gift from Anat Chengyu Liang's lab, USC	DMEM+10%FBS
cell line (homo sapiens)	U251	Other	Gift from Anat Erdreich-Epstein's lab, USC	DMEM+10%FBS
cell line (Cercopithecus aethiops)	Vero	ATCC	CCL-81	DMEM+10%FBS
primary cell (human)	primary human epidermal keratinocytes	GIBCO	C0015C	EpiLife Medium+EDGS
Antibodies (mouse monoclonal)	HPV16 L1 Antibody (MD2H11)	Santa Cruz Biotechnology	sc-65713	IB:1:2000
Antibodies (mouse monoclonal)	HPV16 L2 Antibody (2JGmab#5)	Santa Cruz Biotechnology	sc-65709	IB:1:500
Antibodies (human monoclonal)	HPV16 L2 Antibody JWW-1	PMID: 25972404	Addgene	IB:1μg/ml, IF:10μg/ml
Antibodies (mouse monoclonal)	β-Actin Antibody (C4)	Santa Cruz Biotechnology	Sc-47779	IB:1:3000
Antibodies (mouse monoclonal)	FLAG	Sigma	F1804	IB:1:3000
Antibodies (mouse monoclonal)	Anti-Human p230	BD	611280	IF:1:500
Antibodies (goat polyclonal)	Anti-Human IgG (Fc specific)−HRP	Sigma	A0170	IB:1:5000
Antibodies (rabbit polyclonal)	Anti-mouse IgG (Whole molecule)−HRP	Sigma	A9044	IB:1:5000
Antibodies (Donkey polyclonal)	Donkey anti-Mouse IgG (H+L), A488	Thermo scientific	A21202	IF:1:1000
Antibodies (Goat polyclonal)	Goat anti-Human IgG (H+L), A568	Thermo scientific	A21090	IF:1:1000
Antibodies (Goat polyclonal)	Goat anti-Human IgG (H+L), A633	Thermo scientific	A21091	IF:1:1000
commercial assay or kit	MagStrep "type3" XT	IBA Lifesciences	2-4090-002	
commercial assay or kit	Pierce™ Protein A/G Agarose	Thermo Scientific	20422	
commercial assay or kit	QUANTI-Luc™	Invivogen	rep-qlc1	
commercial assay or kit	Click-iT Plus EdU A555 Imaging Kit	Thermo Scientific	C10638	
commercial assay or kit	5-Ethynyl-2′-deoxyuridine (EdU)	click reaction tools	1149-100	Working concentration: 20 μM
commercial assay or kit	Hoechst	Thermo Scientific	33342	
commercial assay or kit	OptiPrep™ Density Gradient Medium	Sigma	D1556	
commercial assay or kit	BioLock Biotin blocking solution	IBA Lifesciences	2-0205-250	Working concentration: 70mU/ml
commercial assay or kit	Amersham ECL chemiluminescent substrate	GE	RPN2232	
chemical compound, drug	Bafilomycin A1	Enzo Life Sciences	BML-CM110-0100	Working concentration: 100nM
chemical compound, drug	γ secretase inhibitor-XXI	EMD Millipore	565790	Working concentration: 250nM
chemical compound, drug	Aphidicolin	Sigma	A0781	Working concentration: 3 μM
chemical compound, drug	Furin inhibitor I(dRVKR)	EMD Millipore	344930	Working concentration: 1 μM
chemical compound, drug	Ftase inhibitor II	Santa Cruz Biotechnology	sc-221633	Working concentration: 1 μM
chemical compound, drug	GGTase inhibitor 2133	Santa Cruz Biotechnology	sc-221668	Working concentration: 1 μM
software, algorithm	Vector NTI	Thermo Scientific		
software, algorithm	WebLogo	open sourse		https://weblogo.berkeley.edu/logo.cgi
software, algorithm	Graphpad Prism	GraphPad		
software, algorithm	SnapGene	SnapGene		
software, algorithm	Flowjo	FlowJo, LLC		
software, algorithm	Photoshop CS5	Adobe		
